# Notochordal Cell-Based Treatment Strategies and Their Potential in Intervertebral Disc Regeneration

**DOI:** 10.3389/fcell.2021.780749

**Published:** 2022-03-14

**Authors:** Frances C. Bach, Deepani W. Poramba-Liyanage, Frank M. Riemers, Jerome Guicheux, Anne Camus, James C. Iatridis, Danny Chan, Keita Ito, Christine L. Le Maitre, Marianna A. Tryfonidou

**Affiliations:** ^1^ Department of Clinical Sciences, Faculty of Veterinary Medicine, Utrecht University, Utrecht, Netherlands; ^2^ UMR 1229-RMeS, Regenerative Medicine and Skeleton, Université de Nantes, ONIRIS, Nantes, France; ^3^ UFR Odontologie, Université de Nantes, Nantes, France; ^4^ PHU4 OTONN, CHU Nantes, Nantes, France; ^5^ Leni and Peter W. May Department of Orthopaedics, Icahn School of Medicine at Mount Sinai, New York, NY, United States; ^6^ School of Biomedical Sciences, The University of Hong Kong, Hong Kong SAR, China; ^7^ Orthopaedic Biomechanics, Department of Biomedical Engineering, Eindhoven University of Technology, Eindhoven, Netherlands; ^8^ Department of Orthopedics, University Medical Centre Utrecht, Utrecht, Netherlands; ^9^ Biomolecular Sciences Research Centre, Sheffield Hallam University, Sheffield, United Kingdom

**Keywords:** intervertebral disc – degeneration, conditioned media (CM), secretome, low back pain, extracellular matrix (ECM), cell therapeutic potential, Notochordal cell

## Abstract

Chronic low back pain is the number one cause of years lived with disability. In about 40% of patients, chronic lower back pain is related to intervertebral disc (IVD) degeneration. The standard-of-care focuses on symptomatic relief, while surgery is the last resort. Emerging therapeutic strategies target the underlying cause of IVD degeneration and increasingly focus on the relatively overlooked notochordal cells (NCs). NCs are derived from the notochord and once the notochord regresses they remain in the core of the developing IVD, the nucleus pulposus. The large vacuolated NCs rapidly decline after birth and are replaced by the smaller nucleus pulposus cells with maturation, ageing, and degeneration. Here, we provide an update on the journey of NCs and discuss the cell markers and tools that can be used to study their fate and regenerative capacity. We review the therapeutic potential of NCs for the treatment of IVD-related lower back pain and outline important future directions in this area. Promising studies indicate that NCs and their secretome exerts regenerative effects, via increased proliferation, extracellular matrix production, and anti-inflammatory effects. Reports on NC-like cells derived from embryonic- or induced pluripotent-stem cells claim to have successfully generated NC-like cells but did not compare them with native NCs for phenotypic markers or in terms of their regenerative capacity. Altogether, this is an emerging and active field of research with exciting possibilities. NC-based studies demonstrate that cues from developmental biology can pave the path for future clinical therapies focused on regenerating the diseased IVD.

## Introduction

More than 80% of the human population experiences low back pain (LBP) at least once in their life (O’Sullivan et al., 2019). In 20% of patients who experience LBP, chronic LBP (LBP) may develop. LBP has been the leading cause of years lived in disability since 1990 and is ranked as the condition with the third highest burden of disease, after cardiovascular and cerebrovascular diseases (Wu et al., 2020). With such high prevalence and morbidity, LBP is an ailment with severe socioeconomic consequences. While the underlying cause of LBP is often non-specific, intervertebral disc (IVD) degeneration (IVDD) is the most common one (Luoma et al., 2000; Cheung et al., 2012).

Current first-line treatments for IVDD-induced LBP are multimodal and consist of lifestyle changes, physiotherapy, and analgesic medication. Surgery is a common last option, entailing either removal of herniated material during microdiscectomy and/or decompression, IVD removal with intervertebral fusion, or IVD prosthetic replacement, all with variable success in symptom management and considerable patient morbidities (Malik et al., 2013; Fujii et al., 2019). To manage pain, up to 30–60% of LBP patients are prescribed opioids (Kamper et al., 2020), even though there is poor evidence of efficacy, tolerability, and safety of their long-term use for LBP (Bialas et al., 2020). Thus, all current therapeutic approaches aim at symptom reduction (at least pain) and bring about treatment-specific limitations. Therefore, minimally invasive regenerative strategies that promote biological IVD repair are evolving to address this unmet need, by targeting LBP at an earlier stage, and hence delaying or reducing the necessity for surgery.

**FIGURE 1:**
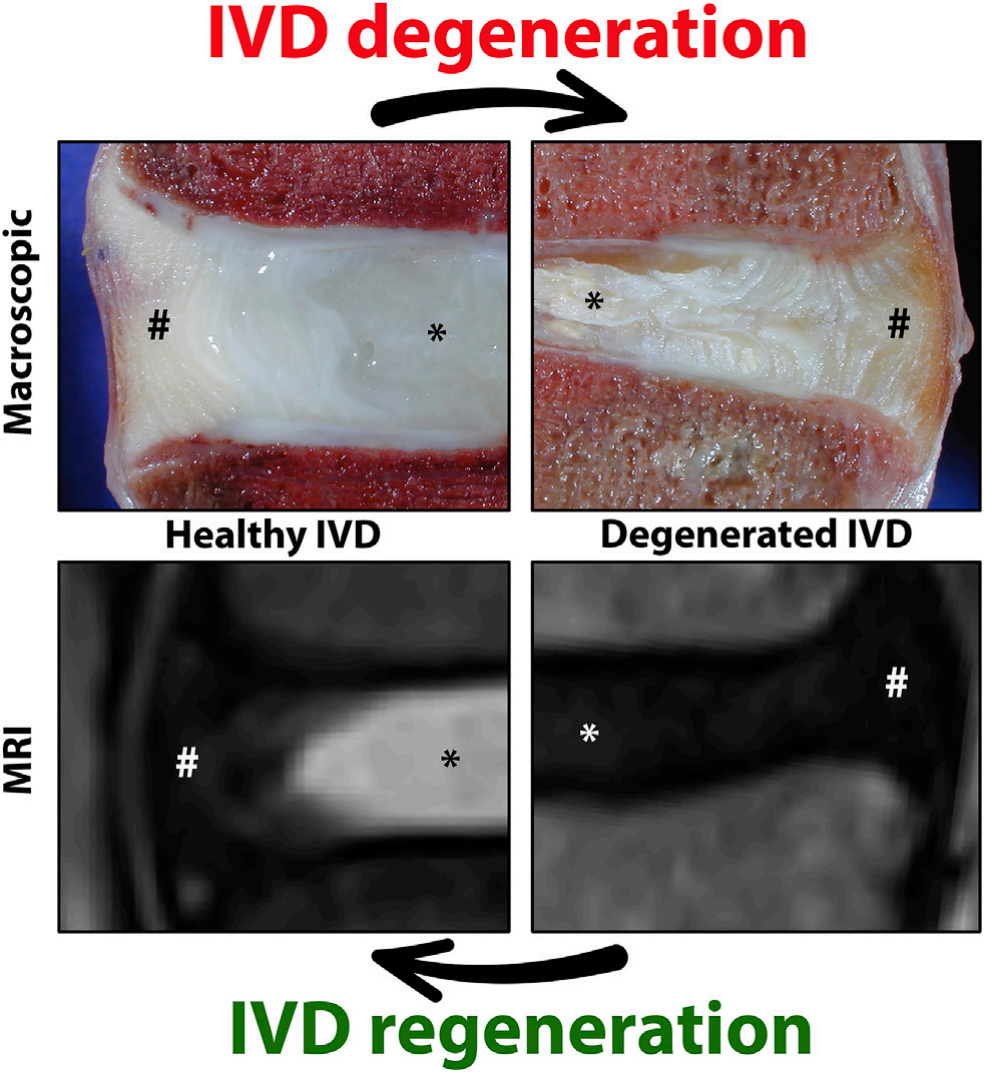
Representation of the healthy and degenerated human intervertebral disc (IVD) at macroscopic and magnetic resonance imaging typically used to visualize the IVD. Human healthy and degenerated L4-L5 IVDs are employed, a location commonly affected in chronic low back pain. The core of the IVD [nucleus pulposus, NP (*)] is constrained by the two endplates and the annulus fibrosus [AF(#)]. A healthy IVD has a gel-like NP with high water and glycosaminoglycan content (white on the T2 weighted sequence). IVD degeneration starts with loss of glycosaminoglycan and water within the NP (black on T2w sequence). Images by courtesy of Prof. Joachim Wilke.

Biological IVD repair should ideally target the underlying degenerative process and induce regeneration of the degenerate IVD, ultimately aiming to restore the biomechanical tissue function to that of a healthy disc, allowing movement, flexibility, and integrity of the spine (Binch et al., 2021). For this reason, IVD regeneration strategies generally target the core of the IVD, the nucleus pulposus (NP), while some strategies targeting the annulus fibrosus (AF) that encapsulates the NP are also being developed (Figure 1) (Gluais et al., 2019; DiStefano et al., 2020; Sloan et al., 2020). Regenerative therapies often focus on cell-based approaches for the degenerate NP targeting underlying causes [reviewed by (Binch et al., 2021)]. Many clinical trials have employed allogeneic mesenchymal stromal cells [MSCs; reviewed in (Barakat et al., 2019)], and report clinical improvement in pain and patient function, however, to date there is no clear evidence for regeneration of the diseased IVD (Loibl et al., 2019). For long-term clinical efficacy in IVD-related LBP, however, it is anticipated that biologic disc repair is essential. An increasing number of studies focus on the relatively overlooked notochordal cells (NCs) residing within the core of the developing disc. The large vacuolated NCs are replaced by smaller nucleus pulposus cells (NPCs) in the IVD during maturation and ageing, which coincides with the onset of degenerative changes, indicating the role of NCs in maintaining tissue health.

**FIGURE 2:**
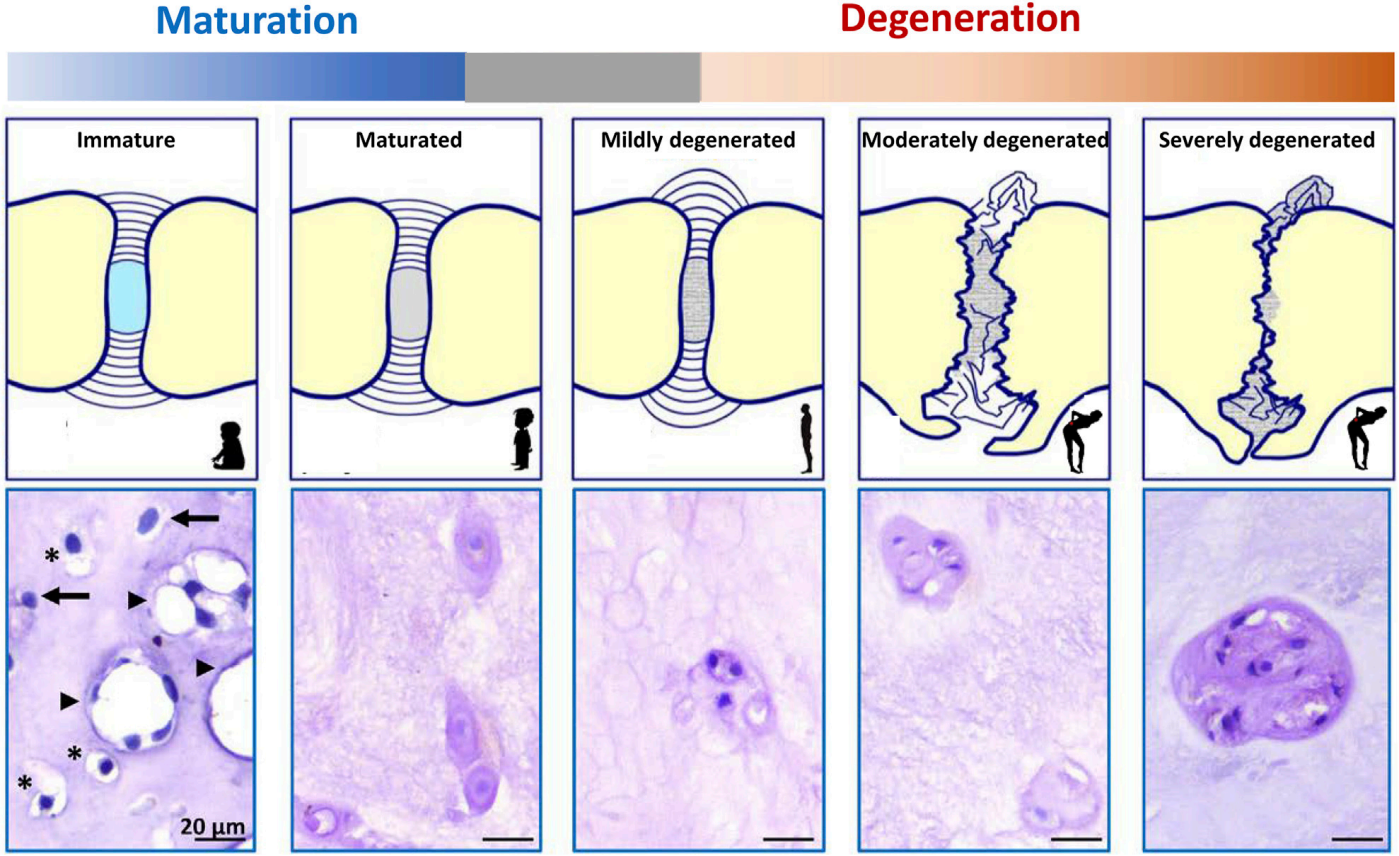
Schematic representation of intervertebral disc (IVD) maturation and degeneration during human life. During IVD maturation, a transition in nucleus pulposus (NP) cell phenotype occurs from large, vacuolated notochordal cells (NCs) to smaller, non-vacuolated nucleus pulposus cells (NPCs). Humans have lost (almost) all their NCs before adulthood. In immature human NPs (*e.g,.* fetuses and babies), vacuolated NCs (arrowhead), transitional cells with some small vacuoles remaining (*), and non-vacuolated NPCs (arrow) or only NPCs are present (dependent on the fetal stage/age of the donor). In maturated human IVDs (*e.g.,* individuals >10 years), only NPCs are present. Degenerated IVDs contain single NPCs, or small or large clusters of NPCs (as a reactive reparative response during moderate/severe degeneration).

In this review, we assess the therapeutic potential of NCs for the treatment of IVD disease-related LBP and outline the important future directions in this evolving area. Given the essential role of NCs, the present review first provides an update on the journey the NCs travel, ending up in the developing and maturing disc and how they evolve with ageing and degeneration (Figure 2) in the human IVD. Within this context, an overview is provided on cell markers corresponding with the phenotypes of the cells residing in the core of the IVD, expanding on tools that will help to better understand their ontology and cell fate mapping, with a primary focus on the regenerative capacities of the NCs.

## The Intervertebral Disc

The IVD consists of cartilaginous endplates (EPs) and AF, which together surround the NP (Figure 1). The healthy NP has a hydrated gelatinous extracellular matrix (ECM), mainly composed of glycosaminoglycans (GAGs) and type II collagen (Bergknut et al., 2013a). Cations are attracted to the negatively charged GAGs and draw water molecules into the NP by osmosis. The hydrated NP has the potential to swell but is restricted by the EPs and AF, which together pressurize the IVD. In this way, together with joint motion and muscle contraction, the IVD dissipates energy and distributes loading and axial compressive forces exerted on the spine (Smit, 2002). The IVD is the largest avascular organ of the body and the cells within the NP receive their nutrition by diffusion through the permeable cartilaginous EPs and the AF. The histology and physiology of the healthy IVD have been extensively reviewed (Pattappa et al., 2012; Sivan et al., 2014; Zeldin et al., 2020); here we focus on the (degenerative) changes of the IVD during maturation and ageing. During IVD ageing, changes occur, which overlap with those seen during maturation. To date, there is a grey area where histopathological changes seen in a subclinical stage reflect reactive degenerative changes (Figure 2; Box 1).

Generally, IVD degeneration is considered to start in the NP, since histologic signs of NP degeneration can already be present before AF tearing exists (Bergknut et al., 2013b). During the first stages of IVD degeneration, the GAG and water content of the NP decreases, while the relative collagen content increases with a switch from collagen type II to type I and increased collagen cross bridges. Altogether this results in less pressurized and more rigid tissue. The degenerate IVD is not able to effectively withstand circumferential loads, and since it cannot adequately repair its ECM, the IVD weakens and is further damaged by physiologic loading (Binch et al., 2021). With a less pressurized NP, the loading is converted to the other components, *e.g.* compression of the AF and bending loads on the EPs. This change in loading direction is considered to be initially responsible for AF tearing, EP fracturing, and/or disc herniation. IVD degeneration is further mediated by the production of cytokines by the disc cells themselves, which drives catabolic processes within the IVD (Phillips et al., 2015; Navone et al., 2017). In the otherwise avascular and aneural IVD, IVD degeneration results in ECM changes and the production of mediators by cells to create a permissive environment for ingrowth of nociceptive nerve fibers and blood vessels. This contributes to pain sensation, together with the production of inflammatory chemo/cytokines (*e.g.* interleukins, neurotrophins, substance P), and mechanical compression of nerve structures (Tolofari et al., 2010; Ito and Creemers, 2013; Navone et al., 2017).

### Cellular Journey From the Notochord

**FIGURE 3:**
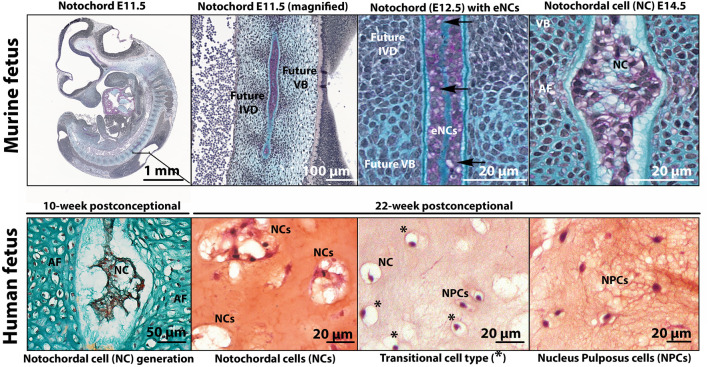
Histologic images to exemplify the terminology of embryonic notochord cells (eNCs), vacuolated notochordal cells (NCs), non-vacuolated nucleus pulposus cells (NPCs), and cells with a transitional phenotype (with some small vacuoles remaining). In E11.5 and E12.5 murine fetuses, the notochord is a rod-like structure with tightly packed small non-vacuolated eNCs confined by the basement membrane sheath (Alcian Blue). In mice, the appearance of small intracytoplasmic vacuoles (arrow) are an indication of eNC into NC transition observed from E12.5. By E14.5, the regression of the notochord is almost complete and the intervertebral discand vertebral bodies are being formed (Alcian Blue). At this stage, NCs exhibit large intracytoplasmic vacuoles. In contrast, a 10-week postconceptional human fetus (Safranin O/Alcian Blue), the notochord is pinching into NP tissue containing NCs, and the rest of the IVD (annulus fibrosus (AF) and vertebras) are being formed. The human NC, transitional cell type, and NPC (Safranin O/Fast Green) pictures were obtained from a 22-week postconceptional human fetus. Human NC generation image by courtesy of Wilson Chan and Vivian Tam; murine images by courtesy of Maeva Dutilleul. E, embryonic day; IVD, intervertebral disc; VB, vertebral body.

While all components of the IVD are of mesodermal origin [reviewed by (de Bree et al., 2018; Séguin et al., 2018)], the NP is the only component that represents remnants of the notochord, the transient embryonic midline structure that defines all vertebrates. Fate mapping experiments in mice were the first to demonstrate that as the embryonic axial skeleton develops, the notochord is progressively dismantled in areas where the vertebral bodies form, but expands in areas that later form the NP (Choi et al., 2008). The different cells present within the NP (from conception towards adulthood) come with many different names and consensus in the field is lacking. To describe the key moments of cellular transition, and in the absence of nomenclature in this field, this review section introduces the complex terms surrounding the transition of embryonic notochordal cells (eNCs; residing within the notochord) towards NCs to NPCs. The vocabulary employed for cells residing in the NP from development to adulthood is explained in Box 2 with corresponding examples in Figure 3.

Notochordal development is observed in human embryos between Carnegie stages 7–12 (15–30 days of embryonic development) (de Bakker et al., 2016; de Bree et al., 2018). The notochord is essentially a rod comprised of a core of large, vacuolated eNCs surrounded by a thick basement membrane sheath composed of collagen, laminins and proteoglycans (Choi and Harfe, 2011) (Figure 3). eNCs have been extensively studied in the zebrafish: each eNC essentially gives rise to a single fluid-filled vacuole that occupies the entire cell volume (Bagwell et al., 2020). It is this retention of the hydrated material within the large vacuole while being confined by the notochordal sheath that attributes the structural properties of the notochord, allowing for notochord expansion in an anteroposterior axis. As the fetus develops, the formation of the vertebra condenses the notochord into regions where the IVD would later appear (Rodrigues-Pinto et al., 2016). In human embryos from 3.5 weeks postconception, the first appearance of eNCs has been described, while these cells became restricted within the fetal IVD segments after 8–10 weeks postconception (Rodrigues-Pinto et al., 2016). During this later stage, eNCs were completely absent from the vertebral bodies, with just remnants of the notochordal sheath being present in this region. From 10 to 18 weeks postconception, with the trapping of the eNCs within the developing NP, the cells transition into NCs (Rodrigues-Pinto et al., 2016). These NCs retain the vacuoles of the eNCs, but the vacuoles become increasingly fragmented when compared to the large, non-fragmented vacuole of eNCs. NCs show an increased extracellular matrix production capacity compared with eNCs, which is released into the extracellular space. This ECM is rich in GAGs and separates the originally condensed cells of the notochord into cell clusters (Séguin et al., 2018). While the exact mechanisms responsible for the transition of the notochord (eNCs) into NPs (NCs) is unknown, two potential mechanisms have been discussed and include a pressure (during the formation of vertebra, eNCs are pushed into regions where the IVDs are formed) and a repulsion/attraction (eNC movement is driven by attractant/repulsive protein expression, e.g. Eph and/or Robo/Slit pathway) model (Lawson and Harfe, 2015).

**FIGURE 4:**
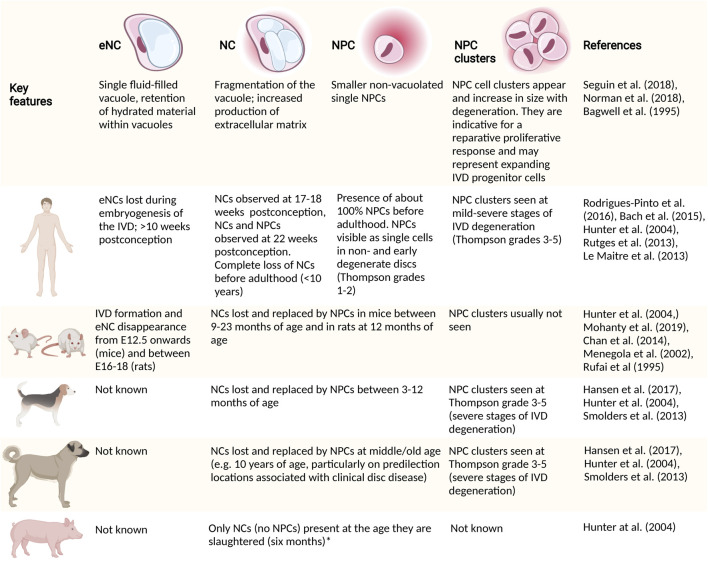
High level differences in embryonic and postnatal cell transitions within the nucleus pulposus (NP). Cellular morphological transitions of the cells in the NP and associated interspecies variation in species commonly used as models to study NC (pathobiology) are provided. Large animal models, including sheep, goats and cows lose their NCs rapidly after birth (Alini et al., 2008). *, Authors have evidence that pigs keep their NCs until at least 2 years of age. CD, chondrodystrophic; E, embryonic day; eNC, embryonic notochord cell; IVD, intervertebral disc; NC, notochordal cell; NCD, non-chondrodystrophic; NP, nucleus pulposus; NPC, nucleus pulposus cell.

As the fetal IVD matures, a transition in NP phenotype is again observed (Figure 4). The large vacuolated NCs gradually disappear, with NC transition towards NPC occurring in human embryos as early as 22 weeks postconception (Bach et al., 2015) (Figure 3), giving way to the appearance of cells with a transitional phenotype. With time, a heterogeneous population of non-vacuolated NPCs, which may possess a variety of phenotypic and functional profiles (Hunter et al., 2004; Richardson et al., 2017) (Figure 2). Human individuals start losing their NCs already before birth (Bach et al., 2015), and their NCs are completely replaced by NPCs at around 10 years of age (Hunter et al., 2004). In other species, this change in cellular phenotype also occurs, but at different ages/life stages (Figure 4). Mature NPs from sheep (Hunter et al., 2004), goats (Daly et al., 2016), and cows (Alini et al., 2008) contain no NCs, while mature NPs from mice (Alini et al., 2008), rabbits (Alini et al., 2008), rats (Alini et al., 2008), and pigs (Hunter et al., 2004) maintain the NC population throughout adulthood. In dogs, NCs are lost at about 1 year of age in chondrodystrophic breeds (e.g., Beagles and Dachshunds), while NCs remain in the NP until middle/old age in non-chondrodystrophic breeds (*e.g.* Shepherds and Mongrels) (Smolders et al., 2013a). Why vacuolated NCs transition towards non-vacuolated NPCs has many reasons, including genetics, considering the differences in NC preservation in breeds within one species (*e.g.* observed in dogs (Smolders et al., 2013a) and mice [Barrionuevo et al., 2006; Bedore et al., 2013; Merceron et al., 2014; Bach et al., 2016a; Choi et al., 2018; Zhang et al., 2018; Tsingas et al., 2020)]. Furthermore, the presence and disappearance of vacuoles is influenced by physicochemical parameters, including osmotic (Hunter et al., 2007; Wuertz et al., 2007; Spillekom et al., 2014; Palacio-Mancheno et al., 2018) and mechanical stress (Purmessur et al., 2013a; Bian et al., 2017), as well as nutritional deprivation (Guehring et al., 2009).

IVD degeneration results in specific changes in the NP**,** with NPC cell clusters dominating the moderately and severely degenerated NP; a clear difference from the single cells seen in postnatal mature and early degenerated discs (Le Maitre et al., 2005). These cell clusters of 4–12 cells are typically considered reactive degenerative changes and have been recently proposed to represent expanding IVD progenitor cells within the degenerate NP (Brown et al., 2018).

### Gene and Protein Expression Analysis of Notochordal Cells

Temporal gene and protein expression changes occur during the transition of the cells residing within the core of the disc. Combining developmental biology studies and fate mapping strategies in mice have highlighted key differences in signaling and cellular biosynthesis associated with each stage of cell transition within the NP [reviewed by (Tessier and Risbud, 2021)]. Since mice maintain their NCs beyond adulthood, direct extrapolation of the information available from mice towards humans is not justified.

In 2014, members of the Orthopaedic Research Society Spine Section recommended a panel of human NP markers including stabilized expression of Hypoxia-inducible factor 1α (HIF-1α), Glucose transporter 1 (GLUT-1), Sonic Hedgehog (SHH), Brachyury (T), cytokeratin (KRT) 18/19, Carbonic anhydrase 12 (CA12), CD24 and an aggrecan/collagen type II ratio >20 (Risbud et al., 2015). Here we review key publications from 2010 to 2020 to compile expression analysis of the human NP (Table 1). Studies attempting to reproduce the aforementioned markers and validate them at the protein level, however, found limited agreement (Thorpe et al., 2016). To date, there are no specific markers that can uniquely and exclusively identify the cells of the human NP, at any stage of development. Key contributions into NP biomarkers at the protein level have mainly used immunostainings. While immunostainings identify NP markers at single-cell resolution, it is inherently biased by the effects of tissue processing, and the availability, choice, and cross-reactivity of antibodies and could thereby also possibly explain contradictory reported findings.

**TABLE 1:** Expression markers reported on human specimens that may define the journey of embryonic notochord cells (eNC) into notochordal cells (NCs) residing within the disc, eventually losing their vacuolated phenotype and becoming non-vacuolated nucleus pulposus cells (NPCs).

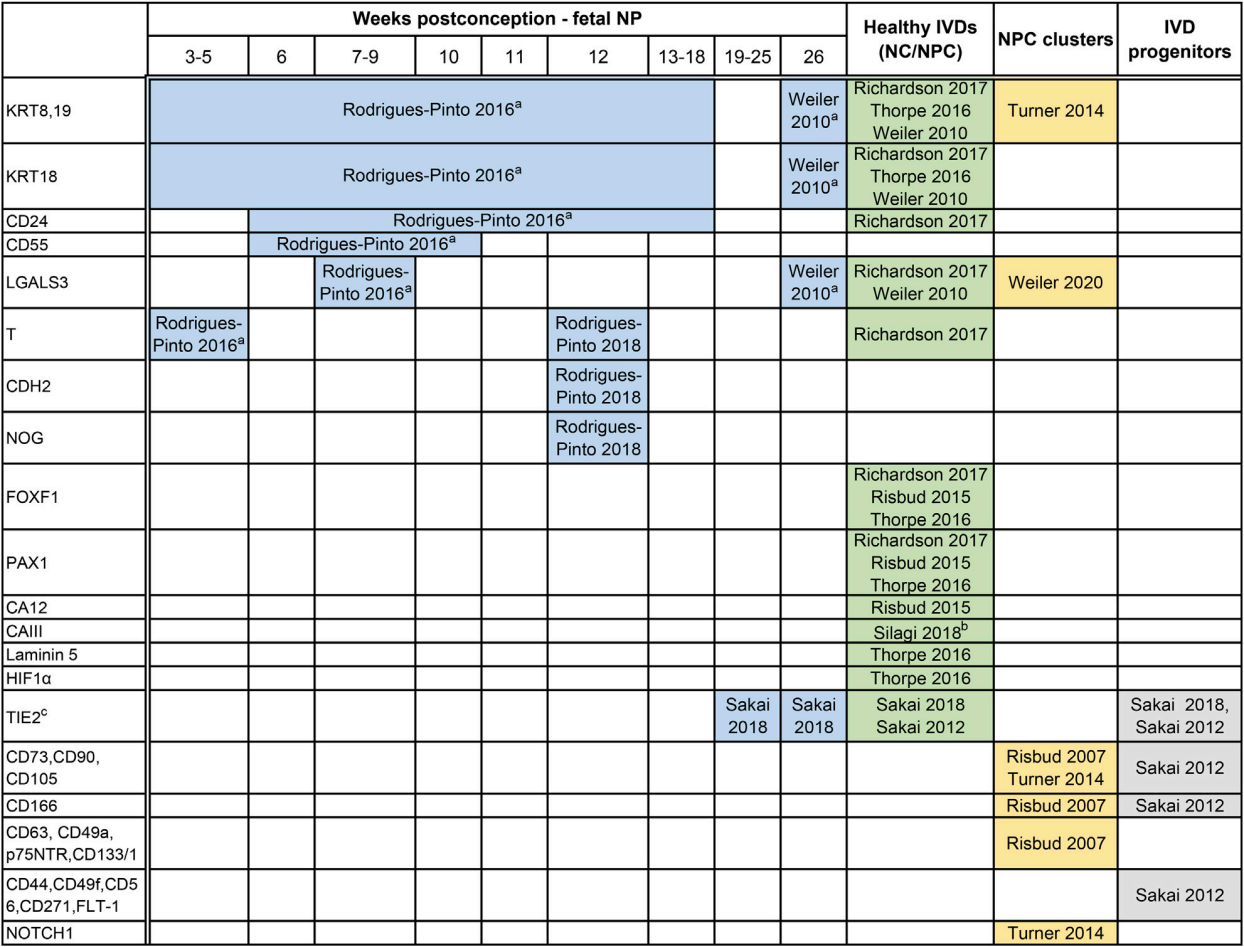

Based on immunostainings, markers for the cells residing within the notochord (<8–10 weeks postconception) and the core of the intervertebral disc (IVD), the nucleus pulposus (NP) have been plotted based on the following life stages: a) human fetal notochord, b) fetal NP, tissue, and c) postnatally, in healthy discs after birth until 25 years of age (macroscopical grading Thompson score 2). Furthermore, markers studied in relation to stemness have been provided to demonstrate possible overlap, including a) NP cell clusters that typically represent reactive degenerative changes, but may also relate to an attempt for repair, and b) NP progenitor cells with replicative capacities. The majority of these markers is expressed higher in the NP,than in the annulus fibrosus (AF). Of note, immunopositivity is largely dependent on the antibodies employed and tissue processing methods. CA, carbonic anhydrase; CD, cluster of differentiation; a CD; cell surface markers typical of stem cells; CDH2, N-cadherin; FLT-1, flotillin-1; FOX, forkhead box; GD2, disialoganglioside 2; GLUT, glucose transporter; HIF1α, Hypoxia-inducible factor 1α; KRT, cytokeratin; LGALS3, galectin-3; NOG, noggin; NOTCH1, Notch homolog 1; p75NTR, p75 low-affinity growth factor receptor; PAX1, Paired Box 1; T, brachyury; TIE2, tyrosine-protein kinase receptor.

a Specific immunostaining in the NP at specific life stages while undetectable in AF or endplates.

b Undetectable in the AF.

c TIE2 + GD2–CD24– cells (TIE2 single-positive cells) behave like dormant stem cells that switch into TIE2 and GD2 double-positive (TIE2 + GD2 + CD24– Cells) active stem cells, with higher expression of the MSC markers, CD44, CD49f, CD56, CD73, CD90, CD105, CD166, CD271 and flotillin-1 (FLT-1). The stem cells then transition in to GD2+ single positive, NP cell progenitors and finally to CD24 single positive NP cells.

From a practical standpoint, NP-specific markers can broadly be differentiated depending on the age of the tissue of the harvested NP cells: markers that are expressed by a) cells from the notochord (eNCs), b) cells in fetal NP tissue (fetal NP markers), c) healthy postnatal tissue from birth until 25 years of age, including tissue graded up to the macroscopic Thompson score of 2, and d) degenerate NP tissue with Thompson score ≥3. To date, it is impossible to discern between markers of maturing NPCs or NPCs undergoing early degenerative changes. There are markers that overall decrease with ageing and degeneration, like KRT8/18/19 (Weiler et al., 2010). Depending on the age of the donor, there are scarcely studied markers that allow for identification of eNCs/NCs from the AF [carbonic anhydrase (CA) III (Silagi et al., 2018)] and/or EP (galectin-3; LGALS3) (Rodrigues-Pinto et al., 2016). Furthermore, there are, probably due to technical challenges that come with isolation of these cells from human fetuses and postnatal donor samples, contradicting findings reported on KRT19, CD90, and TIE2. KRT19 was proposed to be an NP-specific marker for embryonic discs (Rodrigues-Pinto et al., 2016) and was later shown to be co-expressed by the AF cells in young adult discs (Thorpe et al., 2016). Furthermore, while CD90 and TIE2 were undetectable on NP immunostainings (Rodrigues-Pinto et al., 2016); TIE2 was later shown to be present in human fetal NP tissue via immunostainings and flow cytometry, while CD90 appeared universally expressed in TIE2+ or TIE2-human NP cells (Sakai et al., 2018). A unique subset of NP progenitor cells (NPPCs) that shares some similarities with typical MSC markers was also identified (Risbud et al., 2007; Sakai et al., 2012; Brown et al., 2018). The transition of NPPC towards mature NP cells with the concurrent expression of surface markers has been studied in detail. Given the intrinsic reparative capacity, declining with ageing and degeneration, NP cell clusters in the human degenerate IVD have also been studied and assumed to originate from progenitors (Turner et al., 2014; Brown et al., 2018). To date, it is unclear how the NPPCs relate to NCs, other than that NCs can also express TIE2 (Sakai et al., 2018).

From a fundamental perspective, defining the heterogenous NP cell population via single-cell analysis, will assist in assessing the regenerative capacity of the different subpopulations and define which phenotypes are the most potent. This set of markers can be further enriched by discoveries in NC studies in the species that preserve them [*e.g.,* markers that demonstrate the presence of vacuoles, like the aquaporin transmembrane channel proteins previously shown in canine NCs (Snuggs et al., 2019)], but will need to be confirmed on human tissues. From a clinical perspective, such sets of markers are essential in characterizing and benchmarking the NC-based therapies, to the healthy NP cells and also compare them to the standard of care currently in the clinic (*e.g.,* MSCs) and NPPCs.

### Single-Cell Studies

With the available set of markers as a start, follow-up studies employing emerging technologies will fill the gap in NP markers. While mRNA profiling gives an unbiased assessment of marker expression, bulk analysis of the heterogenous NP cell population often falls short in defining the NP phenotypic profile. Recent advances in single-cell technologies have improved the resolution of experiments from the tissue to the cellular level, finally opening up the possibility to address cellular heterogeneity. This is particularly interesting for the scarcely available human tissues during embryonic development and maturation of the disc.

Thousands of single cells can be studied in one experiment; yet, this sheer volume of data makes analysis and statistical interpretation a challenge (Angerer et al., 2017; Jew et al., 2020). Additionally, data from single-cell analysis remain noisy compared to bulk RNA-seq (Jew et al., 2020) and is further challenged by the overlap and asynchrony between the developmental stages of a cell, as shown in blood development (Angerer et al., 2017). These challenges in single-cell data analysis and interpretation need to be taken into account when reviewing the limited single-cell studies available to date from human IVD tissues (Fernandes et al., 2020; Gan et al., 2021). While single-cell analysis is just gaining momentum in the field, Fernandes et.al (2020) was the first to analyze transcriptomes of human NP and AF cells at a single cell level of a limited number of cells that were expanded before profiling. However, as culture *in vitro* is known to affect the cell phenotype, profiling expanded cells may not necessarily reflect the native NP cell profile. Gan et al., 2021 elegantly analysed single-cells from NP, AF and EP of five healthy human postnatal IVD samples (Pfirrmann grade I, from four donors aged 16–31). The analysis was able to focus on nine prominent cell clusters in the IVD tissues analyzed, with three clusters of SOX9+ expressing NPCs, coexpressing the ECM genes, COL2A1 and ACAN, broadly classified into three functional patterns, regulatory, homeostatic and hypertrophic on gene expression. A smaller, NC cluster with high T brachyury expression along with cytokeratins (KRT8) was identified in the NP, together with an NPPC cluster marked by the expression of the mesenchymal progenitor cell markers, PDGFRA and PRRX1, and growth factor IGF1 were identified among the clusters. The low number of NCs identified is in line with the decline of NCs in the human IVD with age, and is possibly a reflection of the Pfirrmann grade I samples from the relatively young donors included in the analysis. Further, single-cell isolation techniques that rely on steps to strain away bigger cell clusters may entrap the larger vacuolated NCs, further hindering the identification and processing of the larger vacuolated NCs. These studies also highlight the difficulty of obtaining NC containg IVD samples of sufficient quantity and quality for these single-cell studies. Despite minor setbacks, the ability to assemble a detailed and multidimensional view of each cell, within the context of throusands of other cells gives the opportunity create a comprehensive cellular map of the IVD. Ultimately, these results question the notion of a unique “cell type”. When looking at the complex landscape of single-cell transcriptomics, what exists are diverse, transient, and plastic cell behaviors, indicating that we should view the cellular composition of the NP beyond the current rigid cell landscape.

### Notochordal Cell Ontogeny Models

#### Fate Mapping Strategies

While single-cell transcriptomics has just become popular in the field of IVD biology to deconvolute the cell types within the heterogeneous populations of the NP, another way to determine NP phenotypic markers is to follow the cells as they differentiate. In mice, fate mapping strategies together with whole transcriptome analysis have been key to exploring differential marker expression associated with different stages of cell transition within the NP. NP of young and adult mice (<1 year of age) mainly consist of NCs (Winkler et al., 2014; Mohanty and Dahia, 2019; Mohanty et al., 2019). Notochord-specific lineage-tracing studies often make use of tissue-specific-inducible Cre-based reporters to trace the fate of eNCs (Chan et al., 2014). Previous work based on the SHH-Cre (Choi et al., 2008; Peck et al., 2017), NOTO-Cre (McCann et al., 2012), and the tamoxifen-inducible SHH-CreERT2 (Choi et al., 2008) and KRT19-CreERT (Mohanty et al., 2020) mouse models indicate that all cell types within the NP of mice are derived from the embryonic notochord. Studies on aged mice are rare and scarce work indicate that the NC to NPC transition occurs between 9 months 1 and 2 years of age (Winkler et al., 2014; Mohanty and Dahia, 2019; Mohanty et al., 2019).

Another attractive strategy is the use of reporters to select specific cells at different stages of differentiation. These fate-mapping studies can only be done in mice, but are instrumental as exploratory studies to find markers that can later be verified in human IVD tissue. For instance, cells isolated from SHH-Cre; ROSA:YFP mice at E12.5 and postnatal day 0 can be employed to investigate specific cells populations that represent eNC to NC transformation (Peck et al., 2017). Significant SHH pathway expression was demonstrated in eNCs, while the transforming growth factor-β (TGF-β)/insulin-like growth factor pathway, and ECM expression was significantly higher in NCs (Peck et al., 2017).

#### Mouse Models

Such strategies can be further enriched by using mouse models to study IVD degeneration (Table 2), which can provide insight into the role of genetics and specific genes in NCs towards NPC transition, as well as the use of live-cell imaging technology to monitor this transition (Joo Han Kim et al., 2009). These fundamental and mechanistic studies involving mice will not only shed light on NC (patho)physiology but will also assist in further identifying and confirming cell markers for the specific life-stages of NCs; its journey from the notochord to the disc and its role during maturation and ageing. With the identification of the different NP cell phenotypic profiles and the ability to isolate the targeted cell populations becoming more achievable, the following sections highlight the advantages of using NCs for the treatment of IVD degeneration-related LBP.

**TABLE 2:** Mouse models and genes shown to play a role in preservation of the notochordal cell phenotype (in alphabetical order).

Mouse model	Phenotype
ACAN^CreERT2^SOX9^fl/fl^ mice	Transcription factor SOX9 conditionally deleted in aggrecan expressing cells. Progressive degeneration of the end plates and extracellular matrix remodeling in both the NP and AF, consistent with human disc degeneration Tsingas et al. (2020). SOX9 null discs showed the presence of non-vacuolated NP cells Tsingas et al. (2020), while conditional SOX9 deletion results in NC death in developing notochord Barrionuevo et al. (2006).
CAV1 null mice	The NP of <6 month old WT mice is rich in viable NCs, whereas the NP of CAV1 null mice mainly contains NPCs and increased NP cell death at already 1.5 months of age Smolders et al. (2013b); Bach et al. (2016a). *Relevance to other species:* In humans and canines, NCs also express high levels of CAV1 Smolders et al. (2013b); Bach et al. (2016a).
CTGF null mice	Notochord specific CTGF loss disrupted the IVD phenotype in embryonic until aged mice Bedore et al. (2013). Although not stated, the histological examples provided appear to demonstrate that NPCs had replaced NCs in the NP of 12- and 17-month old CTGF null mice, whereas NCs were still present in WT murine NPs Bedore et al. (2013), implying that CTGF is essential for NC preservation.
HIF-1α null mice	Progressive disappearance of the NCs from E15.5, concurrent replacement with a novel tissue that resembles fibrocartilage Merceron et al. (2014). This process resembles the changes usually observed during human IVD degeneration.
SM/J mice	Poor regenerative healing capacity; early onset IVD degeneration compared with LG/J mice (good healers) Choi et al. (2018); Zhang et al. (2018). In SM/J, the presence of NPCs is already observed at 2 weeks of age Choi et al. (2018); Zhang et al. (2018). Contrary, LG/J mice maintain a relatively constant pool of NCs during the first months of life Choi et al. (2018); Zhang et al. (2018).

ACAN: aggrecan, AF: annulus fibrosus, CAV1: caveolin-1, CTGF: connective tissue growth factor, NC: notochordal cell, NP: nucleus pulposus, NPC: nucleus pulposus cell, SOX9: SRY-Box Transcription Factor 9, WT: wild type.

BOX 1 **Terminology of Intervertebral Disc Maturation and Degeneration in Humans**

**IVD maturation** entails transitional changes in the IVD, e.g. decrease in GAG:collagen ECM ratio (and water content) and a change in cellular phenotype of the NP (from NCs to NPCs), that do not yet lead to low back pain (Figure 2). This process already starts before birth (in fetal and juvenile IVDs) in human individuals and precedes the development of IVD degeneration (Hunter et al., 2004), while its starts at a later life stage (adolescence/adulthood) in other species, e.g. dogs, pigs (Alini et al., 2008).

**IVD degeneration** encompasses reactive changes compared to the matured IVD, e.g. further decrease in GAG:collagen ratio and the presence of about 100% NPCs within the NP, but also AF bulging/tearing, EP fracturing and vertebral sclerosis, eventually leading to IVD collapse and/or disc protrusion/extrusion (Figure 2). The large vacuolated NCs disappear in humans during maturation (in early childhood) and the age of onset of IVD degeneration is on average around 30–50 years of age. As such, the presence of non-vacuolated chondrocyte-like cells (NPCs) does not relate with clinical disease. Accordingly, progression in IVD degeneration based on (radiographic) imaging is also seen in asymptomatic individuals, although MRI finding are more prevalent in adults with LBP than controls (Brinjikji et al., 2015).

BOX 2 **Terminology of Cells Residing in the Nucleus Pulposus Cells From Development to Adulthood**

Several cellular (morphological) transitions occur in the core of the intervertebral disc (IVD), the nucleus pulposus (NP), during life, *e.g.* by changes in loading and IVD size. These transitions involve changes in extracellular matrix and cellular phenotype of the NP. Unfortunately, consensus terminology is lacking for defining the cells residing within the NP from development to adulthood. Therefore, in this review the following vocabulary applies: the cells residing in the embryonic notochord are termed embryonic notochord cells (eNCs). Once the IVD has developed, the notochord-derived large, vacuolated cells found in the prenatal fetal/postnatal juvenile NP are termed notochordal cells (NCs). Once these cells transition to smaller non-vacuolated chondrocyte-like cells they are termed nucleus pulposus cells (NPCs) (Figure 3).

The morphology of the nucleus pulposus cells during the different life-stages from health to disease may differ among species. This review focuses on the nucleus pulposus cells of the human IVD. For representative examples of the other species used in disc (patho)biology the reader is referred to the recent series of a multi-species histopathological grading schemes reported for mice (Melgoza et al., 2021), rats (Lai et al., 2021), rabbits (Gullbrand et al., 2021), large animal (Lee et al., 2021), and human (Le Maitre et al., 2021) discs.

**Table:** 

Common terms used in the field	In this review discussed as
*Notochordal cells* Weiler et al. (2010); Merceron et al. (2014); de Bree et al. (2018): cells within the notochord	*eNCs*
*Mature notochordal cells* Colombier et al. (2020) *, Notochordal-like cells* Zhang et al. (2018) *, Notochordal NP cells* Wuertz et al. (2007); Choi et al. (2018) *,* and *NP progenitors* Colombier et al. (2016) *:* the vacuolated cells in the NP	*NCs*
*Chondrocyte-like cells* Bergknut et al. (2013a); Risbud et al. (2015); Rodrigues-Pinto et al. (2016); Bach et al. (2017a); Bach et al. (2017b); Séguin et al. (2018); Zhang et al. (2018); Mohanty et al. (2019) *, Chondrocyte-like mature NP cells* Guehring et al. (2009) *, Mature nucleus pulposus cells* Saggese et al. (2015); Mohanty et al. (2019) *,* and *Nucleopulpocytes* Colombier et al. (2014); Clouet et al. (2019): small, non-vacuolated cells in the NP	*NPCs*
*Notochord cells* Barrionuevo et al. (2006); McCann et al. (2016); Rodrigues-Pinto et al. (2016); Séguin et al. (2018); Mohanty et al. (2019): cells within the notochord AND the vacuolated cells in the NP	*eNCs* or *NCs* (depending on their location: notochord or NP, respectively)
*Nucleus pulposus/NP cells* Sakai et al. (2012); Bedore et al. (2013); Winkler et al. (2014); Liu K. et al. (2015); Brown et al. (2018); Silagi et al. (2018); Tang et al. (2018); Mohanty and Dahia (2019); Calió et al. (2021): the vacuolated and non-vacuolated cells in the NP	*NCs* or *NPCs* (depending on their (non)vacuolated phenotype)

## Notochordal Cell-Based Therapies for Disc Disease

Several cell-based therapies that focus on regeneration of the IVD are at different stages of completion or have already reached the clinics (Binch et al., 2021). Therapies applied intradiscally that have undergone or are undergoing clinical trials are described in different reviews (Thorpe et al., 2018; Clouet et al., 2019). Generally, two main approaches have been tested for disc repopulation efforts; tissue-specific NPCs or mesenchymal stem cells (MSCs). Tissue-specific cell-based trials employ either autologous disc cells from herniated IVD tissue (Tschugg et al., 2016) or allogeneic disc cells from healthy donors (Thorpe et al., 2018). Although NPCs have a certain regenerative potential (Nishimura and Mochida, 1998; Nomura et al., 2001; Meisel et al., 2007; Hohaus et al., 2008), in an autologous approach, NPCs from degenerated and herniated IVDs are expected to be less potent and less suitable for regenerative purposes (Ciapetti et al., 2012). These autologous approaches are hampered by low cell numbers which require extensive expansion before transplantation and have to demonstrate their efficacy in clinical use. To overcome this, many clinical trials employ allogeneic/autologous MSCs (Yim et al., 2014; Law et al., 2019). MSC-based studies report clinical improvement in pain and patient function, but do not exert a regenerative effect on the diseased IVD. This is evidenced by improved visual analog scale (VAS) and Oswestry Disability Index (ODI) scores, but show large placebo effects and absence of structural tissue improvement on MRI (Mesoblast, 2021). For long-term efficacy, however, it is anticipated that disc regeneration is essential to return tissue composition and structure, improve tissue biomechanics of the entire IVD and thereby interrupt the positive feedback of tissue damage and the aberrant biological catabolic/inflammatory response. NC-based approaches may address this gap given their regenerative effects amongst others, which will be discussed in the next sections.

**FIGURE 5:**
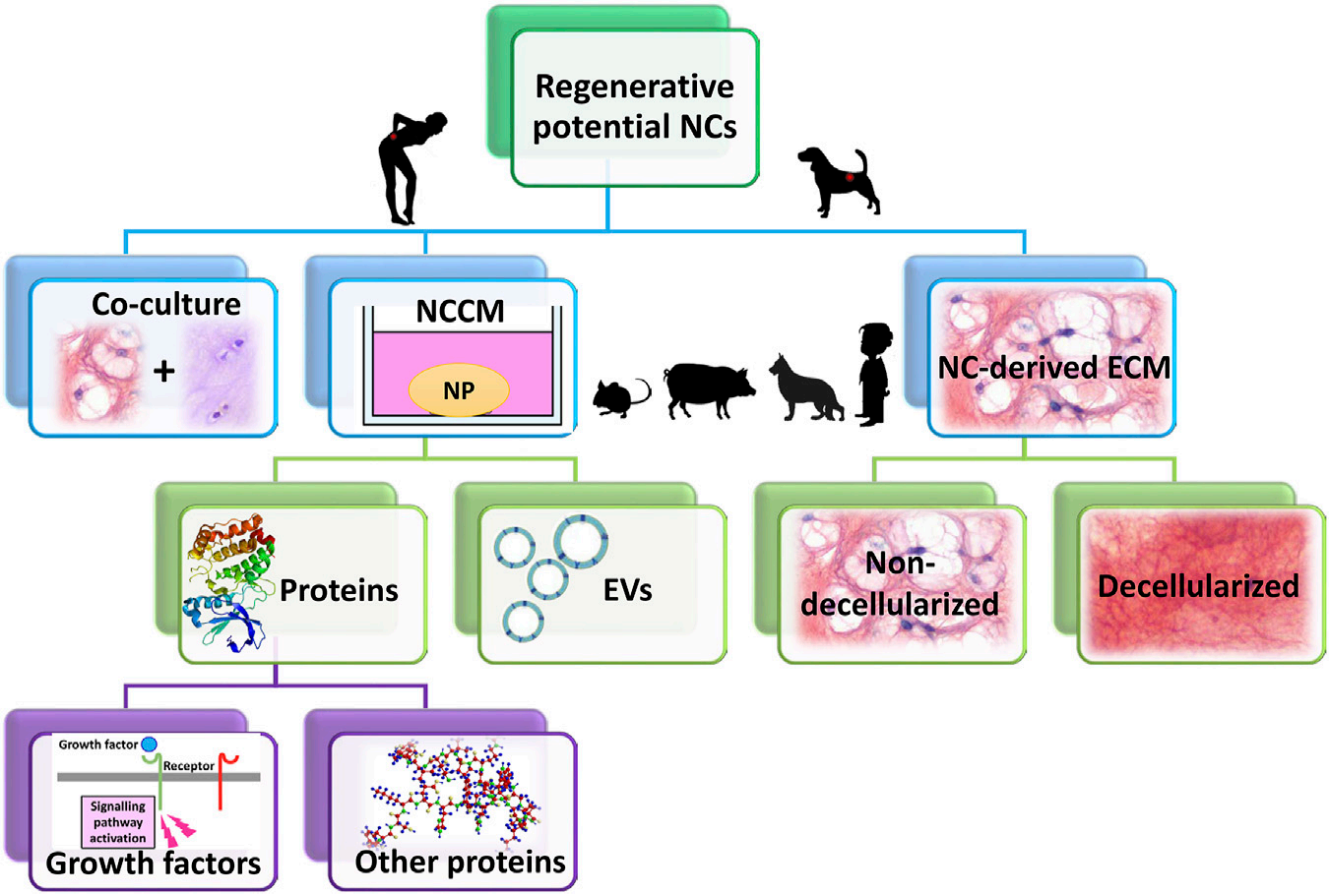
The therapeutic potential of notochordal cells (NCs) has been studied in several ways: either in co-culture with (degenerated) nucleus pulposus cells (NPCs) or by NC-conditioned medium (NCCM). NCCM contains all (bioactive) factors that NCs have secreted, and has been shown to exert regenerative effects on for instance canine and human NPCs (species that suffer from clinical IVD disease). In NCCM, several proteins (growth factors) have been identified, besides extracellular vesicles (EVs). The recent focus of several research groups has shifted from NCCM towards NC-derived extracellular matrix (ECM). This NC-derived ECM (both non-decellularized and decellularized) has been shown to exert regenerative effects, even in *in vivo* canine and rabbit studies. Species that are most commonly used to generate NCCM or NC-derived ECM are small rodents (mice, rats), rabbits, pigs, non-chondrodystrophic dogs. In limited studies, human NC-rich tissues have been employed derived from fetal IVD or from diseased children.

**FIGURE 6:**
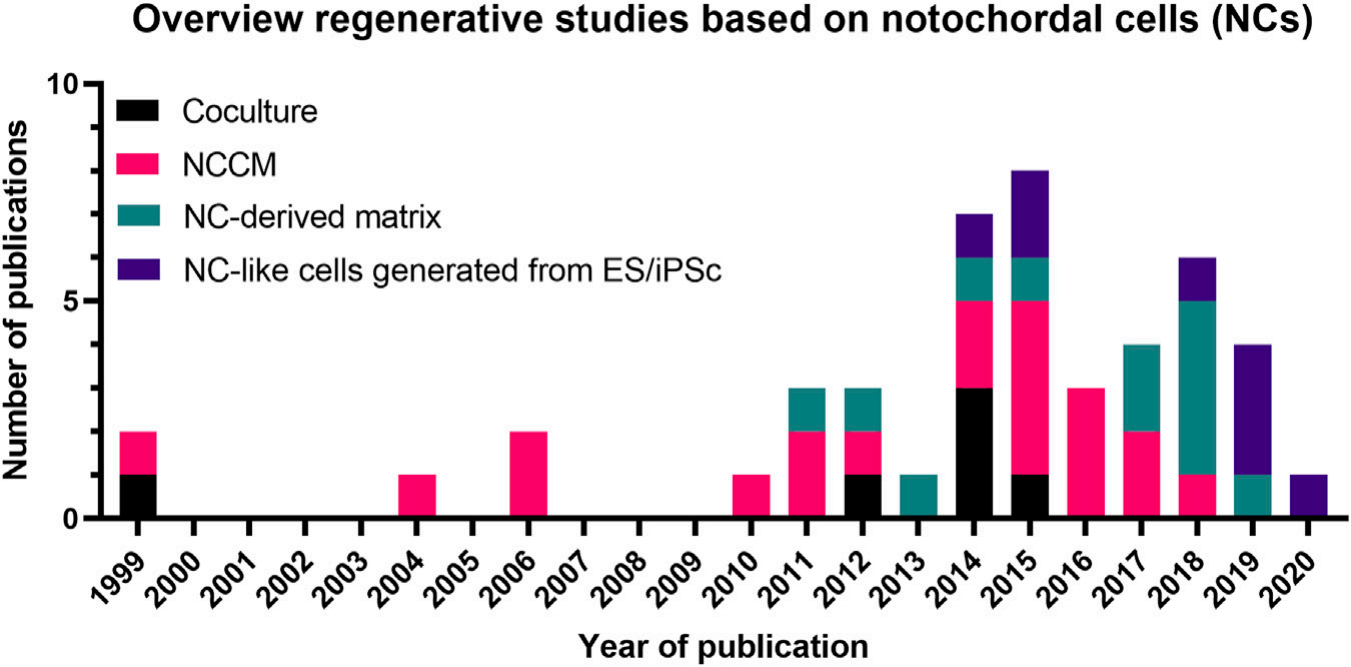
Overview of the number of studies (1999–2021) on the regenerative potential of Notochordal cells. A trend is observed in time from mainly coculture- and NCCM-based studies towards NC-derived matrix and NC-like cell generation studies. Details of these studies are provided in Supplementary Materials S1, S2 and the main text. NCCM, Notochordal cell-conditioned medium; NC, Notochordal cell; ES, embryonic stem cell; iPSC, induced pluripotent stem cells.

The potential therapeutic effects of NCs has been studied in several ways, either in co-culture with (degenerated) NPCs or by NC-conditioned medium (NCCM) (Figure 5). Initially reviewed by Purmessur et al. (2013), an increasing body of literature has since reported further corroborating evidence on the potential of NCs in inducing regenerative (*e.g.* anabolic, anti-catabolic, anti-apoptotic, anti-neurogenic, and anti-angiogenic) effects (Figure 6). Overall, NCs appear to have the potency to rejuvenate the IVD tissue, by increasing NP cell numbers, maintaining healthy NP morphology with high GAG and water content, a loose, flexible collagen type II-rich network, and reduced tissue catabolism, which effectively inhibits neurovascular growth and nociceptive stimuli, thereby reducing pain sensation.

### Co-Cultures of NCs

Co-culture studies generally involve the culture of NC and NPCs together, with the expectation that cell to cell signalling cascades, either *via* contact or *via* secretion of bioactive compounds, can exert a synergistic regenerative response. Aguiar et al. (1999) was the first to show that NCs exerted regenerative effects on NPCs *in vitro* and since then, several studies have confirmed the regenerative potential of porcine and canine NCs on bovine NPCs (Aguiar et al., 1999; Gantenbein-Ritter and Chan, 2012; Gantenbein et al., 2014). However, not all studies could underscore a NC-specific effect due to the absence of control conditions confirming the cell-specific effect (e.g., co-culture of NPCs with other than NCs). However, it is notable that some studies did not observe this regenerative potential of canine and porcine NCs; probably due to changes in NC phenotype in long-term culture under conditions that do not favor their physiology (Potier et al., 2014; Potier and Ito, 2014; Arkesteijn et al., 2015) (see* Preservation of the NC phenotype*). Therefore, NC-conditioned medium (NCCM) offered an alternative to co-culture for studying the therapeutic potential of NCs. More recently, several studies focused on NC-derived ECM.

### Notochordal Cell-Conditioned Medium

To overcome the challenges of cell co-culture, NCCM was employed to study the possible therapeutic effects of NCs. NCCM is typically generated by incubating NC-rich NP tissue in media for a period of up to 4 days, whereafter with the aid of filtering and centrifugation, the conditioned medium is collected (Bach et al., 2015). In the study by Aguiar et al. (1999), besides NC:NPC co-cultures, NCCM was also employed demonstrating that NCs induced GAG synthesis by NPCs independent of cell-cell contact. Since then, more than 20 studies have shown that NCCM (generated from different species, mostly porcine and non-chondrodystrophic canine, under different conditions with different culture media) exerted beneficial effects on various cell types, *e.g.,* NPCs (Aguiar et al., 1999; Erwin et al., 2006; Erwin et al., 2011; Abbott et al., 2012; Gantenbein et al., 2014; Potier et al., 2014; Bach et al., 2015; de Vries et al., 2015; Bach et al., 2016b; Mehrkens et al., 2017), MSCs (Korecki et al., 2010; Purmessur et al., 2011; de Vries et al., 2015), AF cells (Boyd et al., 2004; Gantenbein et al., 2014), (EP) chondrocytes (Ki- Won Kim et al., 2009; Müller et al., 2016) and NP explants (de Vries et al., 2016) (Supplementary Material S1). Importantly, NCCM also exerted positive effects on NPCs from degenerated IVDs of species suffering from clinical IVD disease, *e.g.,* canines (de Vries et al., 2015; Bach et al., 2016b) and humans (Abbott et al., 2012; Bach et al., 2015; Müller et al., 2016; Bach et al., 2017a; Mehrkens et al., 2017). The therapeutic NCCM effects consisted of increased healthy NP-like ECM synthesis (Aguiar et al., 1999; Boyd et al., 2004; Erwin et al., 2006; Erwin and Inman, 2006; Korecki et al., 2010; Erwin et al., 2011; Purmessur et al., 2011; Abbott et al., 2012; Gantenbein et al., 2014; Bach et al., 2015; de Vries et al., 2015; Bach et al., 2016b; de Vries et al., 2016; Müller et al., 2016; Bach et al., 2017a), increased cell proliferation (Erwin et al., 2006; Erwin and Inman, 2006; Bach et al., 2015; de Vries et al., 2016; Müller et al., 2016) and/or decreased apoptosis (Erwin et al., 2011; Mehrkens et al., 2017) (Supplementary Material S1). Intradiscal injection of NCCM even promoted regeneration of degenerated rat NPs *in vivo* (Matta et al., 2017). Other possible beneficial effects of NCCM are the inhibition of angiogenesis (Cornejo et al., 2015) and neurogenesis (Purmessur et al., 2015), although these effects were not reproduced by de Vries et al. (2018). The addition of chondroitin sulphate (a major GAG component of NCCM) alone inhibited vessel formation in HUVEC cells in both studies (Cornejo et al., 2015; de Vries et al., 2018), indicating that chondroitin sulphate from NCCM may be sufficient to prevent neurite and vessel growth, although other factors from NCCM may also be present. Furthermore, it remains to be determined whether and how the effects of NCCM depend on the species, breed, and age of the donor.

Altogether, the factors secreted by NCs exert beneficial effects on many different cell types of relevance to IVD regeneration. The pronounced regenerative effects of NCs on NPCs imply that NCs play a vital role in maintaining IVD integrity. Therefore, NCs are considered a promising target for regenerative and/or symptom modifying therapies for IVD disease. The mechanism of how NCs exert their trophic effects, however, remains to be further elucidated. Several studies have been conducted that provide possible clues on the mode of action of NCCM; these include bioactive proteins secreted by NCs (see section *Proteins Identified in NCCM, Proteins Identified in the Matrisome, Notochordal Cell-Derived ECM and Decellularized NP Tissue*) and the exchange of bioactive factors via NC secreted extracellular vesicles (EVs) (see section *NC-Secreted Extracellular Vesicles*).

#### Proteins Identified in NCCM

**FIGURE 7:**
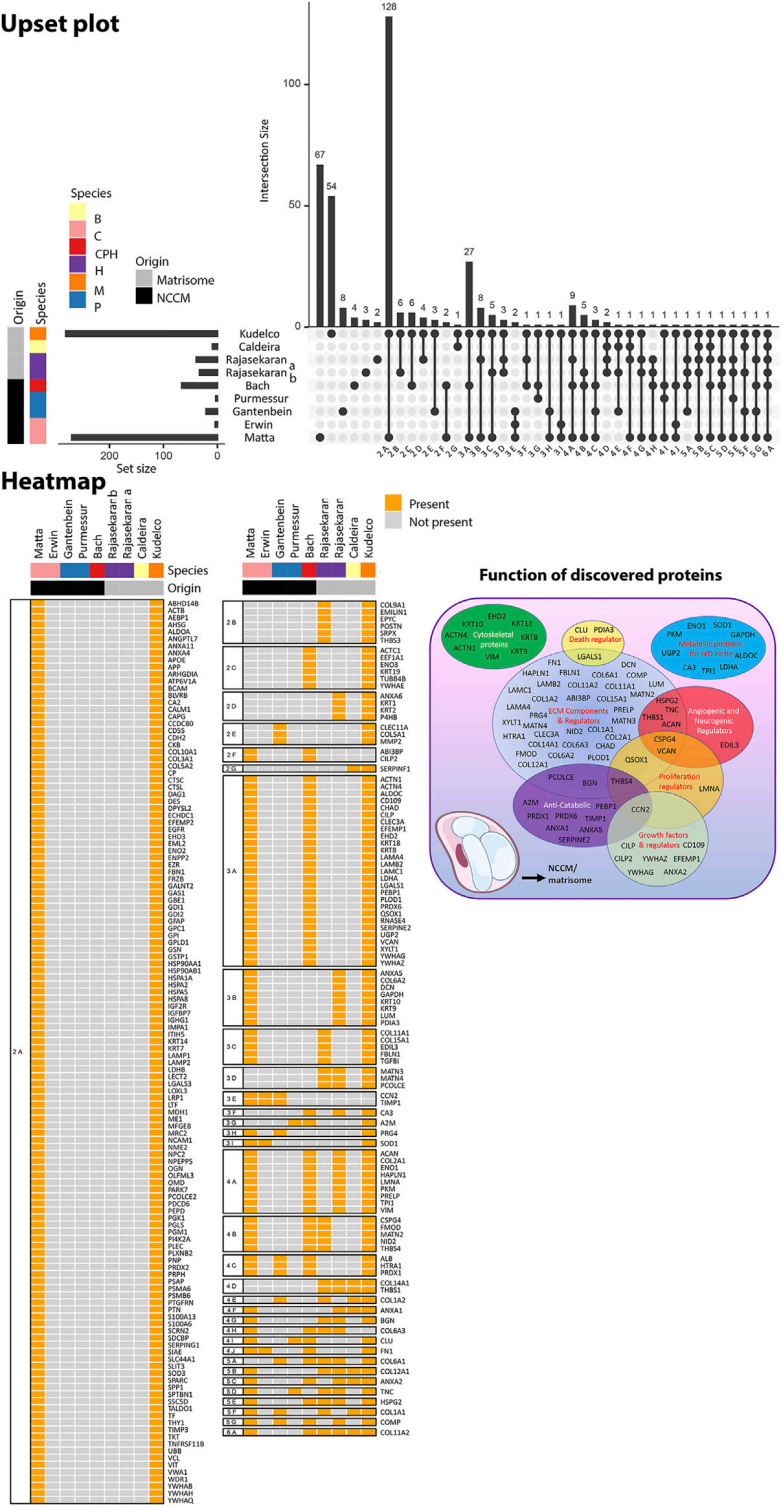
Overview of the proteins that were identified in human, canine, and/or porcine NCCM and murine, human and bovine fetal NC-rich matrisome. The Upset plot depicts the number of proteins identified in the specific study and relates to the number of proteins that overlap with other studies. Intersection size indicates the number of proteins identified in each specific comparison. The set size indicates the number of proteins identified in the specific study. The heatmap depicts the overlap of proteins between the different studies. The main functions of the discovered proteins are provided in a Venn diagram. Purmessur et al. (2011) and Gantenbein et al. (2014) identified proteins in porcine NCCM, while Erwin et al. (2006) and Matta et al. (2017) used canine NCCM. In the study of Bach and de Vries et al. (2016), only proteins that were identified in both human, canine, and porcine NCCM were included in this analysis. Rajasekaran et al. (2020), Rajasekaran et al. (2020b) identified proteins in human fetal matrisome, Caldeira et al. (2017) in bovine fetal matrisome, and Kudelko et al. (2021) in murine matrisome. The study by Tam et al. (2020) was not included in this comparative proteomic overview focusing on NC-rich tissues, since it used NPC-containing adolescent human NP tissue.

NCCM contains the NC-secreted factors, either soluble or ECM-bound, as well as ECM components that are released due to tissue remodeling during culture. To date, five studies focused on the identification of (bioactive) proteins present in NCCM (Erwin and Inman, 2006; Purmessur et al., 2011; Gantenbein et al., 2014; Bach et al., 2016b; Matta et al., 2017) (Figure 7). Based on these studies, it appears that NCCM contains many proteins primarily associated with ECM, anti-catabolism, and growth factors (Figure 7), while upstream analysis identified possible transcription factors involved. The detected matricellular proteins reflect the matrisome produced and remodeled by the NCs. Several of those (including clusterin, tenascin, and α2-macroglobulin) have been suggested to have protective effects for NP cells (Tortorella et al., 2004; Purmessur et al., 2011). In line with this thought, decellularized ECM derived from passaged porcine NCs induced proliferation and chondrogenic ECM production in porcine synovium derived MSCs, indicating that this microenvironment favors differentiation into an NP-like phenotype (Pei et al., 2012).

The NC secretome, rich in ECM molecules, is deposited in the form of a GAG-rich matrix in the NP tissue and brings up challenges in detecting growth factors. Bach et al. (2016) by employing NCCM in proteomic analysis did not detect growth factors. Growth factors secreted by NCs may be overshadowed by large quantities of ECM proteins released by the NP tissue during culture. Furthermore, growth factors may sequestrate within the robust GAG content. The study of Matta et al. (2017) fractionated non-chondrodystrophic canine NCCM by size-exclusion and identifoied that upto 31% of the identified proteins were ECM-related. Several growth factors were also detected, and these include connective tissue growth factor (CTGF, also known as CCN2), TGF-β, Wnt-induced soluble protein 2, insulin-like growth factor binding protein-7, angiopoietin-like 7, and their modulators (chordin, sclerostin and cartilage intermediate layer protein) (Matta et al., 2017).

Within the context of the mode of action of NCs, CTGF is a multifunctional growth factor that interacts with other growth factors such as TGF-β and bone morphogenetic proteins (BMPs), thereby mediating ECM interactions (Figure 7). Furthermore, CTGF can independently induce ECM synthesis (Nakanishi et al., 2000) and plays a role in apoptosis (Gygi et al., 2003; Croci et al., 2004). Pathway analysis demonstrated that the signaling pathways TGFβ, Wnt/β-catenin, axonal guidance, nitric oxide synthase, leucocyte extravasation signaling, and inflammation associated matrix degradation were significantly covered (Matta et al., 2017). Complementary upstream analysis of detected proteins may allow for the identification of potential binding sites and transcription factors involved in NC physiology, including activator protein 1 (AP-1) and paired box gene 4 (PAX4) (Bach et al., 2016b). Furthermore, linking to the possible anti-angiogenic and anti-neurogenic effects (Cornejo et al., 2015; Purmessur et al., 2015), class 3 semaphorins (SEMA) were identified in proteomic analysis of canine (Matta et al., 2017) and porcine (Bach et al., 2016b) NCCM. SEMA3C plays a role in the innervation and vascularization of degenerated human IVDs and has been linked to LBP (Binch et al., 2015). The finding of class 3 semaphorins in canine and porcine NCCM, supports the inhibitory role of semaphorins in nerve and blood vessel growth (Bagnard et al., 1998).

What is the ideal combination of bioactive (growth) factors that can be employed to harness the therapeutic potential of NCs? Anecdotally, only specific growth factors (CTGF and TGF-β) were selected for further analysis given their pivotal role in regulating the expression of several ECM proteins and interaction with other growth factors (Matta et al., 2017). Combinatory treatment with TGF-β_1_ and CTGF restored a healthy, NC-rich NP in a preclinical rat model with induced IVD degeneration (Matta et al., 2017). Also, a single intra-discal injection into degenerated canine IVDs resulted in increased disc height, expression of healthy ECM proteins, and reduced expression of inflammatory mediators (Matta et al., 2018). However, the study did not look into the possible enhancement of tumor development or tissue fibrosis (Bach et al., 2017b; Chen et al., 2020; Zhao et al., 2020). As for an ideal combination for bioactive factors, the key consideration will be to maximize the positive effects of detected bioactive factors in the NCCM, while minimizing their detrimental effects.

#### Proteins Identified in the Matrisome

The NCCM-based proteomic studies observed a plethora of possible (bioactive) components and signaling pathways involved in disc physiology. These possible candidates were recently confirmed in proteomic analysis of human fetal NP tissue at 24 weeks of gestation (Rajasekaran et al., 2020a; Rajasekaran et al., 2020b), bovine fetal NC-rich matrisome at 7 months of gestation (Caldeira et al., 2017) and young murine matrisome (Kudelko et al., 2021) where mainly ECM-related proteins were found (Figure 7). In contrast, the matrisome of NC- and NPC-containing adolescent human NP tissue was distinct from the NC rich matrisome; while these NC low matrisomes contained many ECM-related proteins, they contained even more non-matrisome proteins (Tam et al., 2020). Taken together, these studies shed light on factors important for the composition of healthy NC-containing NPs, but since the fetal tissues of these species, even at these early stages, may have contained NPCs besides NCs (Alini et al., 2008; Bach et al., 2015), the results cannot be confirmed as NC-specific.

#### Notochordal Cell-Derived ECM and Decellularized NP Tissue

Challenged by the complexity of pinpointing the hub-bioactive factors secreted by NCs that exert essential disease-modifying effects, the ECM of NC-rich tissue, the so called NC-derived matrix (NCM), has recently become a focus of attention. It is being considered as a potential candidate, or at least a model system, because of both the bioactive factors it contains, secreted by NCs, as well as replenishing the NP ECM constituents. NCM may, when applied as clinical therapy, comparable to demineralized bone matrix that is widely used in bone regeneration (Gruskin et al., 2012), serve as an ‘instructive matrix’ by locally releasing growth factors and in this way facilitate tissue repair (Bach et al., 2018). Therefore, intradiscal injection of NCM could be a promising regenerative treatment for IVD disease, circumventing the cumbersome identification of bioactive NC-secreted substances or the ethical and regulatory issues raised by the use of NCs in cell-therapy approach (see, *Ethics*).

Recent studies suggested that partially digested rabbit NC-rich NP tissue co-cultured with human NPCs increased ECM production, proliferation, T and KRT18 expression (Bai et al., 2017), indicating that NC-derived NP tissue indeed has regenerative properties (Supplementary Material S2). Furthermore, porcine NCM increased the DNA and GAG content of bovine NPCs *in vitro* (de Vries et al., 2018). In this study, the NCM concentration in the medium was adjusted to obtain a similar protein concentration as NCCM (∼0.4 mg/ml) to allow for comparison. Interestingly, NCM’s anabolic effect on adult human NPCs was stronger compared with NCCM derived from the same porcine spines. In another *in vitro* study, however, 2 mg/ml NCM did not exert any anti-angiogenic and anti-neurogenic effects (de Vries et al., 2018b). Based on the overall promising anabolic *in vitro* benefits, the effect of 10 mg/ml NCM (concentration based on the dose-finding *in-vitro* pilot on canine NPCs) was tested *in vivo* on mildly (spontaneously) and moderately (induced) degenerated chondrodystrophic canine IVDs (Bach et al., 2018). NCM injected in moderately degenerated canine IVDs exerted beneficial effects at a macroscopic level and on MRI, induced type 2 collagen-rich ECM production and improved the disc height. Furthermore, NCM exerted anti-inflammatory effects in bovine NPCs *in vitro* (de Vries et al., 2018) and ameliorated local inflammation in the canine IVD degeneration model *in vivo* (Bach et al., 2018). Although these results look very promising, when translated towards the clinic, one of the bottlenecks that need to be addressed is the decellularization of the NCM to avoid host versus xenogeneic graft responses.

In parallel, promising studies have been conducted with decellularized porcine NCM to support cell-based translational studies (Supplementary Material S2). With this decellularized NCM, human NPC viability was maintained and GAG synthesis *in vitro* was enhanced (Wachs et al., 2017). Furthermore, human MSCs cultured on decellularized porcine NCM expressed NP-cell markers and deposited GAGs and collagens (Mercuri et al., 2011; Mercuri et al., 2013; Zhou et al., 2018; Xu et al., 2019). In the latter study, the cultured constructs of NCM that were seeded with MSCs counteracted IVD degeneration in a rabbit model (Xu et al., 2019). Similar findings were also reported for decellularized porcine NCM combined with rabbit MSCs that partly restored the ECM content of degenerated rabbit NPs *in vivo* (Zhou et al., 2018). Xu et al., 2019 (Xu et al., 2019) also analyzed the MSC-NCM tissue constructs by mass spectrometry and detected the presence of abundant important signaling molecules, *e.g.* TGF-β_1,_ which is in line with the observations from the upstream analysis of proteins detected in NCCM, indicating the involvement of transcription factors mediating TGF signaling (Bach et al., 2016b) Affirmatively, the *in vitro* results of this study were (partially) mediated by increased TGF-β Smad2/3 signaling (Xu et al., 2019)_,_ albeit functional inhibitory studies to confirm this observation is still lacking.

Decellularization is necessary to allow the use of porcine NCM for clinical application. Different decellularization methods have been employed to remove the NCs from the tissue and maintain the ECM. (Supplementary Material S2) Nonetheless, the GAG and collagen content of the decellularized constructs was negatively affected by the decellularization processes in all studies. Since cellular components have numerous ligand-binding domains important for cell-ECM interactions, specific associations with cytokines, chemokines, morphogens, and growth factors that regulate cell differentiation and proliferation (Caterson, 2012), decellularization could affect these bioactive factors; impacting the instructive role of the ECM. Therefore, optimal decellularization agents and protocols remain to be established for this specific application, balancing the safety and maximum instructive capacity of the NCM. Within this context, while fundamental research focuses on the mode of action of NC-based decellularized scaffolds, and follow-up translational work should focus on injectability, dose-finding, and subsequent translation from bench to bedside. Although porcine tissue-derived products have already been clinically applied (Stone et al., 2007; Konertz et al., 2011), safety aspects including methods to remove α-Gal (Park et al., 2009; Wong and Griffiths, 2014) and genetic material [endogenous retroviruses (van der Laan et al., 2000)] without affecting its biologic activity would need to be optimized before the decellularized tissue can be applied clinically in patients. Despite the reduced instructive capacity, decellularized NCM could serve as a cell-free approach or be employed in combination with cell-based therapies. The decellularized NCM could be used as a carrier or even as a primer during the production of the cells to enhance the effects of cell therapy alone.

#### NC-Secreted Extracellular Vesicles

Vesicle related proteins have been identified in porcine, canine, and human NCCM (Bach et al., 2016b) and NC-rich fetal bovine NP matrisome (Caldeira et al., 2017), indicating a role of extracellular vesicles (EVs) in NC biology. EVs are small lipid bilayer-enclosed particles released by cells, that have attracted attention as potential targets for the development of regenerative therapies in the cartilage/IVD field (Malda et al., 2016; Piazza et al., 2020). EVs serve in intercellular signaling, since they are protective carriers for biologically active molecules (*e.g.,* mRNA, miRNA, DNA, protein, lipid) and have the ability to interact with cells in a context-dependent manner (Malda et al., 2016).

Within the context of the IVD, SHH, a key factor in notochord and IVD development, was found to be the cargo of two specific EV fractions from primary chick cells derived from the notochord (Vyas et al., 2014). These two EV fractions contained distinct sets of vesicular miRNAs and proteins with differential effects on the SHH target genes (Vyas et al., 2014), indicating that notochordal EVs play a complex role during embryonic development. A recent study demonstrated that EVs are abundantly secreted by porcine NCs and that these EVs exert anabolic effects upon canine and human NPCs derived from degenerated IVDs (Bach et al., 2017a). Thus, the involvement of EVs within the effects observed by NCCM warrants further investigation. It remains to be determined whether EVs also hold promise for symptom modification, *e.g.,* by inhibiting inflammation, angiogenesis, neurogenesis, or catabolism. Further identifying the bioactive EV-associated cargo, how the micro-environment influences it, the subsets of EVs and their possible distinct biologic effects will provide a better understanding of the NC’s mode of action. This fundamental knowledge will instruct future cell-free regenerative therapies that employ either the native EVs or synthetic nanovesicles delivering specified EV-associated biomolecules (Malda et al., 2016).

## Discussion

NC-based therapeutic strategies have regenerative prospectives to address a yet unmet clinical need for the LBP patient. Next to the scientific advances in NC biology and technology employing their cellular and matrix biology to develop clinically feasible therapeutic strategies, several challenges come with NC-based approaches. These include the bottleneck of maintaining the NC phenotype, ethical considerations with the use of human NCs, and the risk of tumor formation.

### Preservation of the NC Phenotype

From the perspective of employing NCs in cell-based therapies and on the course of fundamental research into their physiology, there are considerable challenges in the preservation of the NC phenotype *in vitro*, since NCs lose their vacuolated phenotype and NC-specific markers during (expansion) culture. Several studies have aimed to optimize the culture conditions for NCs, and the results indicate that NCs retained their morphologic phenotype better by using 3D instead of 2D cultures (Erwin et al., 2009; Smolders et al., 2012; Gantenbein et al., 2014), hypoxic conditions (1–5% O_2_) (Erwin et al., 2009; Omlor et al., 2014; Humphreys et al., 2018), soft (<720 Pa) substrate surfaces (*e.g.,* laminin-coated) (Gilchrist et al., 2011; Fearing et al., 2019; Barcellona et al., 2020) possibly functionalized with ECM-mimetic peptides (Bridgen et al., 2017; Barcellona et al., 2020), increased culture medium osmolarity (from 300 to 400 mOsm/L) (Spillekom et al., 2014), serum-free culture media (Arkesteijn et al., 2016), and low instead of high glucose culture medium (Spillekom et al., 2014; Humphreys et al., 2018). The latter is because NCs are subjected to low glucose concentrations *in vivo* due to the absence of a direct blood supply (Urban et al., 2004). Affirmatively, hyperglycemia has been positively correlated with IVD degeneration in diabetic rats (Mahmoud et al., 2020) and it has been shown that a high glucose environment stimulates apoptosis and inhibits proliferation of NCs *in vitro* (Park and Park, 2013). Altogether, this is a particularly understudied, but essential, field to bring NC-based technologies a step forward within regenerative medicine.

### Ethics

Given that the availability of autologous NPCs (and especially NCs) is extremely low in the adult human IVD (Humphreys et al., 2018), autologous NC treatment is deemed impossible. NCs could be sourced from human specimens (allogeneic; *e.g.,* deceased fetuses-children) with considerable ethical challenges and uncertainties concerning the use of fetal cells/tissues (de Wert et al., 2002; Peng et al., 2020) or from other species (xenogeneic; *e.g.,* porcine). Since the intact IVD is thought to be immune-privileged because of its avascularity (Yang et al., 2009) and the expression of Fas ligand by NPCs, which induces apoptosis of invading Fas-bearing T-cells (Takada et al., 2002; Hiyama et al., 2008), the introduction of allogeneic/xenogeneic cells is not likely to induce a host-versus-graft response. However, even the use of allogenic and xenogeneic cells in regenerative medicine is ethically charged (Niemansburg et al., 2013; Niemansburg et al., 2014) because IVD-related LBP is a disease affecting the quality of life, as opposed to life-threatening, with diseases like heart failure and cancer. As such, the benefits of regenerative medicine-based interventions within the orthopedic field need to be safe and outbalance the probability and possible magnitude of adverse effects, including graft-versus-host disease or tumor formation.

### Risk of Tumor Formation

While research on NCs shows opportunities towards promising therapeutic avenues, there are concerns about their safety. Previous work demonstrated that a few dormant NCs persist in the adult vertebral body and, if activated, may proliferate and give rise to chordomas, which are, however, very rare (McMaster et al., 2001; Bakker et al., 2018). Chordomas contain cells similar to NCs in terms of morphology and express NC-related genes (*e.g.,* T, KRT8/18/19) (Vujovic et al., 2006). Interestingly, although 20% of adult human vertebrae were observed to have NC remnants, they did not develop into tumors (Yamaguchi et al., 2004; McCann et al., 2016). To date, it remains unclear which initiating mechanisms are crucial for the development of chordomas. In line with the minor prevalence of chordomas in humans, in dogs (who typically maintain their NCs throughout life and only lose them at predilection locations to develop LBP) that suffer from variable cancers too, only two cases of histologically confirmed chordomas have been described (Gruber et al., 2008; Stigen et al., 2011). Nonetheless, when aiming for regenerative NC-based treatment strategies, factors that are known to be associated with tumor formation should be carefully investigated.

### Future Perspectives

#### Consensus Terminology

The regenerative potential of NCs is evidenced by the increasing body of literature. NC-based approaches are considered promising for regenerative and/or symptom modifying therapies for LBP due to IVD degeneration. Further efforts to bring NC-based therapies from the bench towards the bedside have to involve alternatives to employing primary NCs. These strategies are hampered by the lack of consensus terminology in defining the cells residing within the NP from development to adulthood (see Box 2), and by the lack of more precise phenotypic descriptions that single-cell technologies are likely to enable in the near future. Such terminology does not only allow for improved communication within the scientific community, most importantly it will assist in defining the specific set of NP cell phenotypic markers. These markers are essential in defining the outcomes of fundamental and translational research. Even more so, these markers will be used to benchmark NC-based therapies being developed.

#### The Role of NC Vacuoles

In the developing notochord, NC vacuoles are considered to be the driving force of axis elongation (Ellis et al., 2013). The NC vacuoles are presumably mechanoprotected by the caveolae, flask-shaped structures of the plasma membrane that express caveolins (Nixon et al., 2007; Sinha et al., 2011; Bach et al., 2016a) and by the ECM of the notochordal sheath, rich in collagens, proteoglycans (mostly chondroitin sulfate and heparan sulfate), laminins, and fibronectins (Yasuoka, 2020). Since the vacuoles play an important biomechanical role in the developing embryo, they might also have a biomechanical role in the postnatal IVD by maintaining intracellular pressure to resist mechanical loading (Hong et al., 2018). Since NC vacuoles are important organelles in the embryonic notochord and postnatal NP, they might also be involved in the NC-mediated regenerative effects. For instance, they may contain bioactive substances in their membrane or vacuole content. During IVD maturation, vacuolated NCs are replaced by non-vacuolated NPCs (Hunter et al., 2003). It is interesting to determine if and how the cargo changes during maturation of the NCs and transition into non-vacuolated NPCs. In addition to the cargo content, also the self-renewal ability of the NCs is a topic of debate. There is evidence that the presence of large intracytoplamic vacuoles is a limiting factor for cell proliferation (Joo Han Kim et al., 2009; Hong et al., 2018), which is important to consider when aiming for NC-based regenerative therapies. Taken together, studying the NC vacuoles may provide novel information, possibly enabling guidance for NC-based IVD regeneration. For this reason, future studies should elucidate the content, composition, and specific role(s) of the NC vacuoles. Lastly, it would be worthwhile studying the exact relationship between the EVs and vacuoles of NCs.

#### Generating NC-Like Cells

Considering the above-mentioned limitation in NC-based approaches, emerging avenues that hold promise involve the generation of NC-like cells from other cell sources, *e.g.,* embryonic (Diaz-Hernandez et al., 2020) or induced pluripotent stem cells (ES and iPS cells) (Liu et al., 2014; Liu K. et al., 2015; Yongxing Liu et al., 2015; Tang et al., 2018; Sheyn et al., 2019; Xia et al., 2019; Colombier et al., 2020; Zhang et al., 2020). Protocols for differentiation into the NC-lineage are highly variable, but generally involve differentiating pluripotent cells towards mesodermal lineages through activation of WNT signaling followed by the transient induction of early embryonic notochordal markers. The published studies do not compare the generated NC-like cells with native NCs in terms of their regenerative capacity or marker expression. While the histologic analysis suggested the presence of vacuolated cells in two studies (Tang et al., 2018; Zhang et al., 2020); their presence needs to be confirmed by markers typically expressed on the membranes of vacuoles. In an *in vivo* study, iPS cell-generated NC-like cells were tested in a porcine model in which degeneration was induced by annular puncture (Sheyn et al., 2019). Although the iPS-derived NC-like cells were detected 8 weeks after injection by KRT18/19, T, and NOTO expression, histology did not show the typical vacuolated NC morphology. Moreover, the GAG or collagen content was not quantified in the treated IVDs, and therefore, regeneration of the porcine IVDs was not confirmed. Interestingly, in a parallel study, porcine NC-derived ECM promoted the differentiation of human iPS cells into NC-like cells (Liu et al., 2014), indicating that modifying the local niche can improve their differentiation. Lastly, the culture conditions optimal for NC preservation (see section *Preservation of the NC phenotype*) can also be used to further optimize the differentiation of pluripotent cells into NC-like cells. Taken together, ES/iPS-based technology to generate NC-like cells can be used for fundamental research to better understand NC development, physiology and regenerative therapies. This is an active field of research with exciting possibilities, but also with ES/iPS-specific and general advanced therapy medicinal product-related bottlenecks and challenges. Examples of ES/iPSC-specific challenges include the improvement of differentiation efficiency, further identification of distinct NC-markers, purification of the differentiated cells, and importantly, maturation into the desired NC phenotype that can thrive and exert therapeutic effects within the hostile degenerative disc environment.

Besides ES or iPS cells, the earlier mentioned local tissue-specific NPPCs (TIE2+ cells) can be considered as an alternative for NC-like cell generation. The relationship between NPPCs and NCs is not clear yet, since NCs can be either TIE2+ or TIE2 negative (Sakai et al., 2018). Moreover, NPPCs can be detected in very young, but also (to a lesser extent) in older human individuals (Sakai et al., 2012), an age at which NCs are not present in the disc anymore. Therefore, NPPCs are considered a specific cell population in the NP, and might not be part of the NC or NPC population *per se*. Also, the fact that NCs have a low (Joo Han Kim et al., 2009), while NPPCs have a high proliferation capacity (Sakai et al., 2012) provides evidence against the hypothesis that NPPCs represent the (entire) NC population. Therefore, TIE2+ NPPCs might be a more suitable tissue-specific cell source for NC-like generation, that might differentiate even more efficiently into the desired cell type than other types of stem cells.

#### Regenerative Effects Beyond the Borders of the Disc

Although this review focuses on LBP due to IVD degeneration, patients with related neck pain (Peng and DePalma, 2018) due to IVD degeneration can probably benefit from NC-based regenerative treatments in the future. Furthermore, during osteoarthritis (OA), the articular cartilage undergoes processes resembling late stages of IVD degeneration, such as hypertrophic differentiation and calcification (Rutges et al., 2010; Rustenburg et al., 2018). Since NPCs closely resemble articular chondrocytes and NCs can induce regenerative effects on NPCs, they may exert positive effects on chondrocytes as well. Confirming this hypothesis, NCCM was demonstrated to exert anabolic and anti-inflammatory effects on human OA chondrocytes (Müller et al., 2016). Moreover, a recent study tested the potency of NC-derived ECM as a bioactive regenerative lubricant for the osteoarthritic joint (de Vries et al., 2018a). This NC-derived matrix exerted anabolic and anti-inflammatory effects on healthy bovine articular chondrocytes and showed promising lubricating properties, comparable to hyaluronic acid (de Vries et al., 2018a). Future studies are warranted to determine the effect of NC-secreted substances on human OA cartilage explants, which may respond differently from (healthy) chondrocytes. Altogether, these two studies show that local application of NC-secreted substances could have potential as a treatment not only for IVD disease but also OA and perhaps even other (orthopedic) diseases.

## Conclusion

The now historic observation that NCs are lost when humans show signs of IVD disease has given rise to many studies suggesting the presence of NCs can have a positive effect on the maintenance of a healthy IVD status. In the last few decades, research into NCs has considerably improved, both qualitatively and quantitatively, with enhanced methods, markers, and more specific scientific questions. Results show promise that NC and their secreted substances (proteins, EVs, and ECM) have a regenerative capacity upon other cell types, such as NPCs. The scarcity of NCs in adult IVDs indicates that regenerative treatments require NC-like cell generation (e.g., embryonic- and induced pluripotent-stem cells) using methods currently being determined and optimized. For future perspectives, however, more efficacy and safety studies are needed, mainly into the long-lasting effects (e.g., tumor formation). In conclusion, NC-based studies demonstrate that by applying the fundamental concepts from developmental biology, the path can be paved for future clinical therapies focused on regenerating the diseased IVD.
