# Supplementary material for: Notochordal Cell-Based Treatment Strategies and Their Potential in Intervertebral Disc Regeneration
Source: Front Cell Dev Biol. 2022 Mar 14;9:780749. doi: 10.3389/fcell.2021.780749 (PMC8963872; doi:10.3389/fcell.2021.780749)
Supplement: Supplementary file 1 [file Table1.DOCX]

Supplementary Material

**Supplementary table 1.** Effects of Notochordal Cell-Conditioned Medium (NCCM) determined *in vitro* and *in vivo* (1999-2021)

| **Study** | **NCCM source (animal)** | **NCCM generation**  **(medium, time)** | **NCCM generation**  **(tissue/cells, culture system)** | **Cells/tissues where the effect**  **of NCCM is determined on** | **Effect of NCCM** |
| --- | --- | --- | --- | --- | --- |
|  |  |  |  |  |  |
| **Aguiar,**  **1999** (1) | * Canine (NCD, *n*=?, age=?); cNCCM | * Hybridoma medium  * 24 hours  * NX | Alginate beads (10,000 cells/bead). Rest unknown | * Bovine adult NPCs (*n*=?, age=?)  * Bovine fetal NPCs (*n*=?, age=?)  * Both: alginate beads (10,000 cells/bead). | * 18 hours: cNCCM induced GAG synthesis |
| **Boyd,**  **2004** (2) | * Porcine (*n*=?, 4-5 months); pNCCM | * OPTIMEM  * 3 days  * NX | Alginate beads (2*10^6^ NCs/mL alginate), 30 beads in 2 mL medium /well (equals 1* 10^6^ cells/well) | * Porcine NCs and AF cells (*n*=?, age 4-5 months) in alginate beads (2*10^6^ cells/mL alginate), 30 beads/well | * 48 hours: pNCCM decreased *COL1*, *COL2* and *ACAN* expression in NP cells. NCCM increased *COL2,* *ACAN* expression in AF cells |
| **Erwin and Inman,**  **2006** (3) | * Canine (NCD, *n*=?, 9-18 months); cNCCM | * DMEM  * 4 days  * NX | Alginate beads (unknown number of NCs/mL alginate); 20, 30, or 40 beads in 2 mL medium/well | * Bovine NPCs ( *n*=3, age=?) in alginate beads (1*10^6^ NPCs/mL alginate), 10 beads in 2 mL medium/well | * 3 days: cNCCM dose-dependently upregulated GAG production and increased cell proliferation (not dose-dependent) |
| **Erwin,**  **2006** (4) | * Canine (NCD, *n*=5, 8-12 months); cNCCM | * DMEM  * 4 days  * NX | * Alginate beads (1*10^6^ NCs/mL alginate) with 120 beads and 2.5 mL medium/well | Bovine NPCs (*n*=?, age=?)  100 alginate beads (rest unknown) | * 24 hours: cNCCM induced *ACAN, VCAN*, *Hyaluronic synthase-2* expression |
| **Korecki,**  **2010** (5) | * Porcine (*n*=5, 2 years); pNCCM | * hgDMEM  * 4 days  * HX | * Alginate beads (2*10^6^ NCs/mL alginate); 1*10^6^ NCs per 5 mL NCCM | * Human BMSC (*n*=3) pellets of 250,00 cells | * 7 days: GAG/DNA content of pNCCM-treated pellets higher than control and TGFβ-treated pellets |
| **Purmessur, 2011** (6) | * Porcine (*n*=8, 5-8 months); pNCCM | * lgDMEM  * 4 days  * HX | * NCA (2*10^6^ NCs/mL alginate), 10 beads in 2 mL medium /well  * NCT (NC-rich NP tissue) à 3 discs (0.9-1.3gram)/30 mL medium | * Human BMSC (*n*=3) pellets of 250,00 cells | *21 days: NCA - downregulated *COL1* and *COL3*, low *COLX*. GAG content not different from control and TGFβ_3-_treated pellets. NCT - GAG content induced and upregulated *COL2* and *SOX9* expression |
| **Abbott,**  **2012** (7) | * Porcine (*n*=?, 2-8 months); pNCCM | * lgDMEM  * 4 days  * HX | * NCA (2*10^6^ NCs/mL alginate), 10 beads in 2 mL medium /well  * NCT (NC-rich NP tissue) à 3 discs (= 1.0 gram) per 30 mL medium  * Both: 20,000 cells/mL NCCM | * Human NPCs (*n*=3, 2*10^6^ NPCs/mL alginate), 10 beads/well, volume NCCM unknown | * 7 days: GAG/DNA content only upregulated by NCA. Matrix gene expression not upregulated in NCA and NCT conditions. *MMP1, MMP3* increased by NTC. |
| **Erwin,**  **2011** (8) | * Canine (NCD, *n*=5-6, 8-12 months); cNCCM  * Bovine (*n*= 5-6, 3 years); BCCM | *ADMEM/F-12  * 3 days  * HX | * Alginate beads (1.5 *10^6^ NCs/mL alginate), 80 beads in 6 mL medium /well  * 2% FBS-supplemented NCCM/ BCCM: 0.75 *10^6^ NCs/mL, 80 beads in 3 mL medium/well | * Bovine NPCs (*n*= 5-6, 3 years) in monolayers of 0.5*10^6^ cells/well | * 48 hours: IL-1β and FasL-mediated apoptosis rescued by cNCCM. Caspase 9 activity decreased by 2%cNCCM and 2%BCCM. Caspase 3/7 activity only reduced by 2%cNCCM. cNCCM increased *ACAN, COL2, ADAMTS4, TIMP1*, and decreased *IL6* and *MMP3* expression. |
| **Gantenbein, 2014** (9) | * Porcine (*n*=4, 4-5 months); pNCCM | * hgDMEM  * 7 days  * HX and NX | * Alginate beads (4*10^6^ NCs/mL alginate); 30 beads in 4 mL medium | * Bovine NPCs (*n*=4, 1 year) in alginate beads, alone or co-culture with porcine NCs.  * Bovine AF cells (*n*=4, 1 year) in alginate beads, alone or co-culture with porcine NCs. | * 14 days: NCs activated NPCs in co-culture and by pNCCM addition (gene expression ratio of *ACAN*/*COL2*↑). AF cells unresponsive to pNCCM. |
| **Potier,**  **2014** (10) | * Porcine (*n*=4, <10 weeks); pNCCM | * hgDMEM  * 4 days  * HX | * 1.0 gram NC-rich NP tissue / 30 mL medium | * Bovine NPCs (*n*=16; 4 pooled per repeat, 22-26 months) in alginate beads (3*10^6^ NPCs/mL alginate)  * Additive effect of bovine BMSC co-culture was also determined. | * 28 days: pNCCM increased NPC proliferation and GAG production to levels similar as TGFβ. NPC:MSC co-culture led to GAG synthesis similar to NPCs alone, which was slightly improved by pNCCM. |
| **Bach,**  **2015** (11) | * Human (*n*=10, 20 weeks of gestation-3 months); hNCCM  * Canine (NCD, *n*=4, 18-23 months); cNCCM  * Porcine (*n*=4, 3 months); pNCCM | * hgDMEM  * 4 days  * HX (all species) and NX (human) | 1.0 gram NC-rich NP tissue / 30 mL medium | * Human NPC (n=3, pooled, 47-63 years) micro-aggregates of 35,000 cells | * 28 days: All species NCCM increased the DNA and GAG content. pNCCM and cNCCM were more potent than hNCCM in inducing GAG deposition. Only hNCCM induced collagen type II production. |
| **De Vries, 2015** (12) | * Canine (NCD, *n*=5, 1-1.5 years); cNCCM | * hgDMEM  * 4 days  * HX | * 1.0 gram NC-rich NP tissue / 30 mL medium | * CD canine NPCs and BMSCs (2-2.5  years) in alginate beads (3*10^6^ NPCs/mL alginate) | * 28 days: cNCCM increased proliferation, GAG production, and expression of genes associated with a healthy NP-like phenotype in NPCs. cNCCM also increased GAG production in BMSCs. When NPCs were co-cultured with BMSCs (in cNCCM), no higher GAG content was observed vs. NPCs alone. |
| **De Vries, 2015** (13) | * Porcine (*n*=10, <3months); pNCCM | * hgDMEM  * 4 days  * HX | * 1.0 gram NC-rich NP tissue / 30 mL medium | * Bovine NP tissue explants (n=5, 2 years, caudal discs)  * Additive effect of 10^6^ bovine BMSCs was also determined. | * 28 days: pNCCM increased GAG content BMSC addition did not increase the GAG or DNA content. |
| **Purmessur, 2015** (14) | * Porcine (*n*=6, 6-8 weeks); pNCCM | * hgDMEM  * 4 days  * HX | * 4 NPs per 30 mL medium  * Filtrate resuspended in an equal volume of either fresh control SH-SY5Y basal media (EMEM: F12K) or rat DRG Basal media (Neuronal Medium + growth serum). | * Human neuroblastoma SH-SY5Y cells and primary rat dorsal root ganglia monolayer culture  * 10% and 100% NCCM tested  * Mechanistic studies included to determine if CS is responsible for NCCM-mediated effects | * 48 hours: pNCCM inhibited neurite outgrowth from SH-SY5Y cells without dose or cytotoxic effects. Neurite growth from SH-SY5Y and DRG cells was not inhibited when cells were treated with pNCCM with digested CS. |
| **Cornejo,**  **2015** (15) | * Porcine (*n*=8, 6-8 weeks); pNCCM | * hgDMEM  * 4 days  * HX | * Amount of tissue per mL hgDMEM unknown  * Filtrate resuspended in Medium 200PRF (Med200) | * HUVEC monolayer culture, *n*=4  * 10% and 100% NCCM tested  * Noggin and CS also tested (notochord is a source of anti-angiogenic factors, *e.g.* noggin, CS) | * 16 hours: endothelial cell invasion inhibited by 10% and 100% pNCCM, 10 and 100 mg CS and 10 and 100 ng noggin  * 24 hours: 10% pNCCM, 10 and 100 mg CS and 10 ng noggin inhibited tubular formation. pNCCM decreased *VEGF-A, MMP-7* and *IL-6* mRNA, CS and noggin did not affect gene expression. |
| **Müller,**  **2016** (16) | * Canine (NCD, *n*=10, 12-18 months); cNCCM | * ADMEM/F-12 with 2% FBS  * Medium collected every 24 hrs, 5 days  * HX | * Alginate beads (1*10^6^ NCs/mL alginate). Rest unknown | Human chondrocyte (non-osteoarthritic; *n*=9 ,18–68 years and osteoarthritis; *n*=6, 60–82 years) in pellets of 2.5 × 10^5^ cells | * 14 days: Healthy chondrocyte pellets recovered GAG content to baseline levels with cNCCM. cNCCM-treated OA pellets increased GAG content and levels of hyaluronic acid link protein, fibromodulin and SOX9. cNCCM reduced IL6, IL-8, MMP-3, MMP-13, and COX-2 expression/secretion. |
| **Bach and de Vries,**  **2016** (17) | * Porcine (*n*=5, 3 months); pNCCM  * Canine (NCD, *n*=8, 16-38 months); cNCCM | * hgDMEM  * 4 days  * HX | * 1.0 gram NC-rich NP tissue / 30 mL medium  * Whole NCCM was also separated into a soluble (NCCM-S; peptides and proteins) and pelletable  (NCCM-P; protein aggregates and extracellular vesicles) fraction | * Porcine NCCM was applied to bovine NPCs (*n*=4 repeats, pooled  2-2.5 years) in alginate beads (3*10^6^ NPCs/mL alginate)  * NCD canine NCCM was applied to CD canine NPCs (CD, *n*=4, 3-10 years) in albumin- and hyaluronic acid containing hydrogels | * 28 days: pNCCM-S exerted a more pronounced anabolic effect than pNCCM-P on bovine NPCs. cNCCM-S exerted a more pronounced anabolic effect than cNCCM-P on CD canine NPCs. pNCCM-P exerted a negligible effect on bovine NPCs. cNCCM-P enhanced GAG and collagen type II deposition by canine NPCs. |
| **Bach,**  **2017** (18) | * Porcine (*n*=7, 1.5 months); pNCCM | * hgDMEM  * 4 days  * HX | * 1.0 gram NC-rich NP tissue / 30 mL medium (whole NCCM)  * Extracellular vesicles (EVs) and proteins were separately isolated from whole porcine NCCM | * Canine NPC (*n*=4, 2-10 years of age, Beagles, pooled) and human NPC (*n*=4, 50-63 years of age, pooled) micro-aggregates of 35,000 cells | * 7 days (canine NPCs): pNCCM-derived EVs induced GAG deposition to a comparable level as pNCCM-derived proteins and whole pNCCM.  * 21 days (human NPCs): pNCCM-derived EVs increased the DNA and GAG content to a lesser extent than whole pNCCM. |
| **Mehrkens, 2017** (19) | * NCD canines (*n*=10, 12-18 months); cNCCM | * ADMEM/F-12 + 2% FBS  * Medium collected every 24 hrs; 3-7days  * HX | * 2-3 NPs per cell strainer within culture wells in 6 mL medium | * Human NPC (*n*=15, 23-80 years) monolayer culture | * 24 hours: cNCCM inhibited cytotoxic stress-induced caspase-9 and -3/7 and maintained mitochondrial membrane potential. |
| **Matta,**  **2017** (20) | * NCD canines (*n*=10, age=8-14 months); cNCCM | * CD Hybridoma media  * 1-2 days  * HX | * 3 NPs were placed within  tissue culture inserts with 40μm-filters in 6 well plates  * cNCCM was centrifuged at 8000 rpm for 30 minutes and filtered through 0.2μm syringe tip filters | * *in vivo*: 12-week old female Wistar rats (*n*= 6 animals/group, 4 discs per animal), disc injury (caudal discs) with 26 G needle  * 4 weeks post-injury: intradiscal injection (~8μL) of concentrated cNCCM (2.2μg/μL) or control medium with 32G needle  * Evaluation after 6 weeks of treatment | * 6 weeks post treatment: NC-rich NPs with moderate Safranin-O staining in cNCCM injected rat-tail injured discs,  * 6 weeks post treatment: increased aggrecan, collagen 2, brachyury, Oct4 and  Nanog protein levels after cNCCM treatment |
| **De Vries, 2018** (21) | * Porcine (*n*=5, 3 months); pNCCM | * hgDMEM  * 4 days  * HX | * 1.0 gram NC-rich NP tissue / 30 mL medium  For HUVEC culture, this material was resuspended in Medium 200 (Life Technologies, Bleiswijk, the Netherlands).  For SH-SY5Y cell culture, the strained material was resuspended in a 1:1 mixture of Eagle’s minimum essential medium (EMEM) and F-12K medium (both from Life Technologies).  * HUVEC culture: filtrate resuspended in Medium  * SH-SY5Y cell culture: filtrate resuspended in an equal volume of SH-SY5Y basal media (EMEM: F12K) | * HUVEC monolayer culture (pool of 10 donors, *n*=5 biological replicates)  * Human neuroblastoma SH-SY5Y monolayer culture (poly-D-lysine coated well plate versus polystyrene culture surface; *n*=5 biological replicates) | * 24 hours (HUVEC): pNCCM induced vessel formation, more matured and organized than basal culture medium. Addition of CS alone decreased vessel growth.  * 24 hours (SH-SY5Y, poly-D-lysine coated surface): pNCCM did not affect the percentage of neurite expressing cells or average neurite length  * 24 hours (SH-SY5Y, polystyrene culture surface): pNCCM increased the percentage of neurite expressing cells |

AF: annulus fibrosus, BMSC: bone marrow-derived mesenchymal stromal cell, CD: chondrodystrophic, CS: chondroitin sulfate, EV: extracellular vesicle, FBS: fetal bovine serum, GAG: glycosaminoglycan, HUVEC: human umbilical vein endothelial cell, HX: hypoxia (1-5% O_2_), IL-6: interleukin-6, NC: notochordal cell, NCD: non-chondrodystrophic, NP: nucleus pulposus, NPC: nucleus pulposus cell, NX: normoxia (20% O_2_), SOX9: SRY-box 9, VEGF: vascular endothelial growth factor

**References**

1. Aguiar DJ, Johnson SL, Oegema TR. Notochordal cells interact with nucleus pulposus cells: regulation of proteoglycan synthesis. Exp Cell Res. 1999 Jan 10;246(1):129–37.

2. Boyd LM, Chen J, Kraus VB, Setton LA. Conditioned medium differentially regulates matrix protein gene expression in cells of the intervertebral disc. Spine (Phila Pa 1976). 2004;29(20):2217–22.

3. Erwin WM, Inman RD. Notochord cells regulate intervertebral disc chondrocyte proteoglycan production and cell proliferation. Spine (Phila Pa 1976). 2006;31(10):1094–9.

4. Erwin WM, Ashman K, O’Donnel P, Inman RD. Nucleus pulposus notochord cells secrete connective tissue growth factor and up-regulate proteoglycan expression by intervertebral disc chondrocytes. Arthritis Rheum. 2006 Dec;54(12):3859–67.

5. Korecki CL, Taboas JM, Tuan RS, Iatridis JC. Notochordal cell conditioned medium stimulates mesenchymal stem cell differentiation toward a young nucleus pulposus phenotype. Stem Cell Res Ther. 2010 Jun 16;1(2):18.

6. Purmessur D, Schek RM, Abbott RD, Ballif BA, Godburn KE, Iatridis JC. Notochordal conditioned media from tissue increases proteoglycan accumulation and promotes a healthy nucleus pulposus phenotype in human mesenchymal stem cells. Arthritis Res Ther. 2011;13(3):R81.

7. Abbott RD, Purmessur D, Monsey RD, Iatridis JC. Regenerative potential of TGFbeta3 + Dex and notochordal cell conditioned media on degenerated human intervertebral disc cells. J Orthop Res. 2012;30(3):482–8.

8. Erwin WM, Islam D, Inman RD, Fehlings MG, Tsui FW. Notochordal cells protect nucleus pulposus cells from degradation and apoptosis: implications for the mechanisms of intervertebral disc degeneration. Arthritis Res Ther. 2011;13(6):R215.

9. Gantenbein B, Calandriello E, Wuertz-Kozak K, Benneker LM, Keel MJ, Chan SC. Activation of intervertebral disc cells by co-culture with notochordal cells, conditioned medium and hypoxia. BMC Musculoskelet Disord. 2014 Dec 11;15:422.

10. Potier E, de Vries S, van Doeselaar M, Ito K. Potential application of notochordal cells for intervertebral disc regeneration: an in vitro assessment. Eur Cell Mater. 2014;28:61–8.

11. Bach FC, de Vries SAH, Krouwels A, Creemers LB, Ito K, Meij BP, et al. The species-specific regenerative effects of notochordal cell-conditioned medium on chondrocyte-like cells derived from degenerated human intervertebral discs. Eur Cells Mater [Internet]. 2015 [cited 2021 Jun 19];30:132–47. Available from: https://pubmed.ncbi.nlm.nih.gov/26388616/

12. de Vries SA, Potier E, van Doeselaar M, Meij BP, Tryfonidou MA, Ito K. Conditioned medium derived from notochordal cell-rich nucleus pulposus tissue stimulates matrix production by canine nucleus pulposus cells and bone marrow-derived stromal cells. Tissue Eng A. 2015;21(5–6):1077–84.

13. de Vries SA, van Doeselaar M, Meij BP, Tryfonidou MA, Ito K. The Stimulatory Effect of Notochordal Cell-Conditioned Medium in a Nucleus Pulposus Explant Culture. Tissue Eng A. 2016 Jan;22(1–2):103–10.

14. Purmessur D, Cornejo MC, Cho SK, Roughley PJ, Linhardt RJ, Hecht AC, et al. Intact glycosaminoglycans from intervertebral disc-derived notochordal cell-conditioned media inhibit neurite growth while maintaining neuronal cell viability. Spine J. 2015;15(5):1060–9.

15. Cornejo MC, Cho SK, Giannarelli C, Iatridis JC, Purmessur D. Soluble factors from the notochordal-rich intervertebral disc inhibit endothelial cell invasion and vessel formation in the presence and absence of pro-inflammatory cytokines. Osteoarthr Cartil [Internet]. 2015 Mar 1 [cited 2021 Jun 19];23(3):487–96. Available from: https://pubmed.ncbi.nlm.nih.gov/25534363/

16. Muller S, Acevedo L, Wang X, Karim MZ, Matta A, Mehrkens A, et al. Notochordal cell conditioned medium (NCCM) regenerates end-stage human osteoarthritic articular chondrocytes and promotes a healthy phenotype. Arthritis Res Ther. 2016 Jun 2;18(1):125-016-1026–x.

17. Bach FC, de Vries SAH, Riemers FM, Boere J, van Heel FWM, van Doeselaar M, et al. Soluble and pelletable factors in porcine, canine and human notochordal cell-conditioned medium: Implications for IVD regeneration. Eur Cells Mater [Internet]. 2016 Jul 1 [cited 2021 Jun 19];32:163–80. Available from: https://pubmed.ncbi.nlm.nih.gov/27572543/

18. Bach F, Libregts S, Creemers L, Meij B, Ito K, Wauben M, et al. Notochordal-cell derived extracellular vesicles exert regenerative effects on canine and human nucleus pulposus cells. Oncotarget. 2017;8(51):88845–56.

19. Mehrkens A, Matta A, Karim MZ, Kim S, Fehlings MG, Schaeren S, et al. Notochordal cell-derived conditioned medium protects human nucleus pulposus cells from stress-induced apoptosis. Spine J. 2017 Apr;17(4):579–88.

20. Matta A, Karim MZ, Isenman DE, Erwin WM. Molecular Therapy for Degenerative Disc Disease: Clues from Secretome Analysis of the Notochordal Cell-Rich Nucleus Pulposus. Sci Rep. 2017;7:45623.

21. de Vries SAH, van Doeselaar M, Meij BP, Tryfonidou MA, Ito K. Notochordal cell matrix: An inhibitor of neurite and blood vessel growth? J Orthop Res. 2018 Jul;
