# Supplementary material for: Notochordal Cell-Based Treatment Strategies and Their Potential in Intervertebral Disc Regeneration
Source: Front Cell Dev Biol. 2022 Mar 14;9:780749. doi: 10.3389/fcell.2021.780749 (PMC8963872; doi:10.3389/fcell.2021.780749)
Supplement: Supplementary file 2 [file Table2.DOCX]

Supplementary Material

**Supplementary Table 2.** Studies using NC-rich NP tissue for IVD regeneration purposes (2011-2020)

| **Study** | **Source (animal)** | **Decellularization** | **Generation** | **Cells/tissue where the effect is determined on** | **Effect** |
| --- | --- | --- | --- | --- | --- |
| **Mercuri, 2011** (1) | * Porcine (*n*=?, age=?) | * 72 h incubation in decellularization solution (50 mM Tris-aminomethane buffer containing 0.1% (w/v) ethylenediamine tetra acetic acid and 0.02% (w/v) sodium azide). Four options were tested:  - 1: 0.15% (v/v) Triton X-100 with 0.25% (w/v) deoxycholic acid  - 2: 0.1% Triton X-100 with 0.15% deoxycholic acid  - 3: 0.05% Triton X-100 with 0.05% deoxycholic acid  - 4: 0.6% Triton X-100 with 1% deoxycholic acid  * Samples incubated in 720 mU/mL DNase + 720 mU/mL RNase (48 h)  * Effect of ultrasonication was tested (10 min periods every 24 h) with option 1 and 4 | N/A | * Human ASCs (*n*=?, age=?), monolayer culture on scaffold, 2.5*10^4^ cells/cm^2^ | * Decellularization was best achieved in combination with ultrasonication (lowest DNA content)  * *in vitro* - 7 days: ASC viability and proliferative capacity were maintained |
| **Mercuri,**  **2013** (2) | * Porcine (*n*=?, age=?) | * Decellularization solution (72 h): 50 mM Tris-aminomethane buffer (pH 7.5) containing 1% (w/v) deoxycholic acid, 0.6% Triton X-100 (v/v), 0.1% (w/v) ethylenediamine tetra acetic acid, and 0.02% (w/v) sodium azide  * 10 min periods of ultrasonication (every 24 h) followed by 720 mU/mL DNase + 720 mU/mL RNase treatment (48 h) prior to 0.1% peracetic acid sterilization (4 h) | N/A | * Human ASCs (*n*=?, age=?), monolayer culture on scaffold, 2*10^3^ cells/cm^2^  * *In vivo* biocompatibility tested in male juvenile Sprague-Dawley rats (*n*=20), scaffold was implanted in subdermal pockets | * *in vitro* - 14 days: ASCs expressed NP-cell markers. GAG and collagen content of ASC-seeded hydrogels increased vs. non-seeded controls  **in vivo* - 28 days: presence of mononuclear cells (e.g. macrophages and fibroblasts), blood vessel infiltration, collagen deposition |
| **Pei,**  **2012** (3) | * Porcine  (*n*=2, 3 months of age) | * After NC monolayer culture reached 90% confluence, 50 μM ascorbic acid was added (8 days)  * Deposited ECM was incubated with 0.5% Triton X-100 + 20 mM ammonium hydroxide (37°C, 5 min) | N/A | * Porcine SDSCs (*n*=2, 3 months of age, 3000/cm^2^) were expanded on ECM deposited by NCs, SDSCs, or NC:SDSC (50:50); thereafter, the expanded SDSCs were cultured in pellets (300,000 cells) in chondrogenic medium (incl. TGF-β_3_) | * 14 days: ECM deposited by NC:SDSCs increased SDSC viability and differentiation toward the NP lineage; this effect is comparable  with ECM deposited by SDSCs but higher than that deposited by  NCs alone |
| **Liu, 2014** (4) | * Porcine (n=?, age=?) | N/A | * Lyophilization, crushed and resuspended in culture medium | * human induced pluripotent stem (iPSC) cell line HDFa-YK26  * Cells were differentiated for 10 days  * Thereafter pellets were generated and chondrogenically differentiated for 28 days | * 10 days differentiated: NP matrix induced iPSCs differentiation towards NC-like cells with expression of NC marker genes (T, CK8, CK18).  * 28 day chondrogenically differentiated: NC-like cells generated NP-like tissue rich in aggrecan and collagen type II |
| **Liu, 2015** (5) | *Porcine (n=?, 2 years of age) | N/A | * Lyophilization, crushed and resuspended in culture medium | * human induced pluripotent stem (iPSC) cell line HDFa-YK26  * Cells were differentiated for 10 days  * Thereafter pellets were generated and chondrogenically differentiated for 28 days | * 10 days differentiated: NP matrix induced iPSCs differentiation towards NC-like cells with expression of NC marker genes (T, CK8, CK18).  * 28 day chondrogenically differentiated: NC-like cells generated NP-like tissue rich in aggrecan and collagen type II |
| **Wachs, 2017** (6) | * Porcine (n=?, age=?) | * NP tissue was immersed in water (7 h), followed by 100 mM sodium and 50 mM phosphate buffer (10 h), SB-10 detergent (4 h), 100 mM sodium and 50 mM phosphate buffer (15 min), Triton X-200/SB-16 (3 h), 100 mM sodium and 50 mM phosphate buffer (3 × 15 min), SB-10 detergent (1.75 h), 100 mM sodium and 50 mM phosphate buffer (15 min), Triton X-200/SB-16 (3 h), 100 mM sodium and 50 mM phosphate buffer (3 × 15 min), and DNase/RNase (75 U/mL/100ug/mL; 34 h) | N/A | * Human NPCs (*n*=?, age=?) were mixed with solubilized decellularized NP samples. The mixture was pipetted in 30 µL droplets (40,000 cells/gel) and incubated (45 min, 37°C) for thermal gelation. Thereafter, the gels were cultured in well-plates | * 21 days: NPC viability was maintained, with morphology similar to native NPCs, and increased GAG deposition |
| **Bai,**  **2017** (7) | * Rabbit (*n*=18, 3 months of age) | N/A | * Two hour digestion with 0.025% collagenase type II | * Human NPCs (*n*=5, 44-53 years of age) cocultured with partially digested NC-rich NP tissue (transwell, 1 · 10^4^ NPCs/well) | * 14 days: increased proliferation, *T* and *KRT18* expression and chondrogenic ECM production |
| **Zhou,**  **2018** (8) | * Porcine (*n*=?, age=?) | * NP tissue was immersed in SB-10 detergent (4 h), Triton X-200/SB-16 (3 h), SB-10 detergent (1.75 h), Triton X-200/SB-16 (3 h), and DNase/RNase (75 U/mL/100 lg/mL; 36 h) | * Lyophilization, crushed and  mixed with CS (1.7 mg/mL),  PBS, ASCs (2.0*10^6^/mL)  * Concentration: 3.5 mg/mL  (based on GAG content before and after decellularization)  * Genipin (0.02% w/v) for subsequent cross-linking | * *In vitro*: Human ASCs (*n*=?, age=?)  * *In vivo*: rabbit ASCs (*n*=5, 4 month old) in rabbit IVD degeneration (puncture model: IVDs were stabbed with 16G needle, depth 5 mm) | * *In vitro -* 7, 14 days: NP-like differentiation of human ASCs  * *In vivo* - 16 weeks: partly regenerated the degenerated rabbit NP |
| **De Vries, 2018** (9) | * Porcine (*n*=12, 3 months of age) | N/A | * Lyophilization, resuspended in plain medium  * Concentration was adjusted to a similar protein concentration as NCCM (~0.4 mg/mL) | * Bovine NPCs in alginate beads (*n*=6, 2-2.5 years of age vs. 4-6 years of age, 3*10^6^ cells/mL alginate)  * Adolescent bovine NPCs were also cultured with 5 ng/mL interleukin-1β | * 28 days: increased DNA and GAG content in adolescent and adult bovine NPCs  * 28 days: NC-rich NP tissue anabolic effect was stronger compared with NCCM derived from the same porcine spines  * 28 days: porcine NC-rich NP tissue exerted stronger effect than bovine NPC-rich NP tissue  * 28 days: anabolic response was observed in an inflammatory environment |
| **De Vries, 2018** (10) | * Porcine (*n*=5, 3 months of age) | N/A | * Lyophilization, resuspended in plain medium (2 mg/mL) | * HUVEC monolayer culture (pool of 10 donors, *n*=5 biological replicates)  * Human neuroblastoma SH-SY5Y monolayer culture (poly-D-lysine coated well plate versus polystyrene culture surface; *n*=5 biological replicates) | * 24 hours: no anti-angiogenic and anti-neurogenic effects observed; on a polystyrene surface, it even induced a higher number of neurite‐expressing cells |
| **Bach,**  **2018** (11) | * Porcine (*n*=6, 3 months of age) | N/A | * Lyophilization, resuspended in plain medium (10 mg/mL) | * Canine (*n*=6, 2–7 years of age) and human (*n*=6, 47–72 years of age) NPC micro-aggregates *in vitro*  *** Canine MSC micro-aggregates *in vitro (n*=3, 4 months - 3 years of age*)*  * Degenerated canine IVDs *in vivo* (*n*=6, 14 months of age) | * *In vitro* - 28 days: increased chondrogenic ECM production of NPCs and MSCs *in vitro*  * *In vivo* - 6 months: beneficial effects at macroscopic and MRI level, induced collagen type II-rich ECM production, improved the disc height, and ameliorated local inflammation *in vivo* |
| **Xu,**  **2019** (12) | * Porcine (*n*=20, age =?) | * After 5 freeze-thaw cycles, NP tissue was immersed in 0.5% SDS for 8 h followed by 2 h 200 U/mL DNAse treatment and flushing in PBS for 12 h to eliminate residual chemicals | N/A | * *in vitro* - Human MSCs (n=?, age=?)  * *in vivo* – Rabbit IVDs (n=?, 6 weeks of age, degeneration induced 4 weeks before treatment injection) | * *in vitro*- 14 days: MSCs seeded in the NP-ECM scaffold differentiated into NP-like cells with aggrecan and collagen type 2 expression due to increased TGF/Smad signaling  * *in vivo* - 8 weeks: decelerated the degeneration of the IVD on MRI |

ASC: adipose-derived mesenchymal stromal cells, CK: cytokeratin, ECM: extracellular matrix, GAG: glycosaminoglycans, h: hours, HUVEC: human umbilical vein endothelial cells, IVD: intervertebral disc, KRT: cytokeratin, min: minutes, MRI: magnetic resonance imaging, MSC: mesenchymal stromal cell, N/A: not applicable, NC: notochordal cell, NCCM: NC conditioned medium, NP: nucleus pulposus, NPC: nucleus pulposus cell, SDSC: synovium-derived stem cell, T: brachyury, TGF: transforming growth factor

**References**

1. Mercuri JJ, Gill SS, Simionescu DT. Novel tissue-derived biomimetic scaffold for regenerating the human nucleus pulposus. J Biomed Mater Res A. 2011 Feb;96(2):422–35.

2. Mercuri JJ, Patnaik S, Dion G, Gill SS, Liao J, Simionescu DT. Regenerative potential of decellularized porcine nucleus pulposus hydrogel scaffolds: stem cell differentiation, matrix remodeling, and biocompatibility studies. Tissue Eng A. 2013 Apr;19(7–8):952–66.

3. Pei M, Shoukry M, Li J, Daffner SD, France JC, Emery SE. Modulation of in vitro microenvironment facilitates synovium-derived stem cell-based nucleus pulposus tissue regeneration. Spine (Phila Pa 1976). 2012 Aug 15;37(18):1538–47.

4. Liu Y, Rahaman MN, Bal BS. Modulating notochordal differentiation of human induced pluripotent stem cells using natural nucleus pulposus tissue matrix. PLoS One. 2014 Jul 23;9(7):e100885.

5. Liu Y, Fu S, Rahaman MN, Mao JJ, Bal BS. Native nucleus pulposus tissue matrix promotes notochordal differentiation of human induced pluripotent stem cells with potential for treating intervertebral disc degeneration. J Biomed Mater Res A. 2015;103(3):1053–9.

6. Wachs RA, Hoogenboezem EN, Huda HI, Xin S, Porvasnik SL, Schmidt CE. Creation of an injectable in situ gelling native extracellular matrix for nucleus pulposus tissue engineering. Spine J. 2017;17(3):435–44.

7. Bai XD, Li XC, Chen JH, Guo ZM, Hou LS, Wang DL, et al. (*) Coculture with Partial Digestion Notochordal Cell-Rich Nucleus Pulposus Tissue Activates Degenerative Human Nucleus Pulposus Cells. Tissue Eng A. 2017 Aug;23(15–16):837–46.

8. Zhou X, Wang J, Huang X, Fang W, Tao Y, Zhao T, et al. Injectable decellularized nucleus pulposus-based cell delivery system for differentiation of adipose-derived stem cells and nucleus pulposus regeneration. Acta Biomater. 2018 Nov;81:115–28.

9. de Vries S, Doeselaar M V, Meij B, Tryfonidou M, Ito K. Notochordal Cell Matrix As a Therapeutic Agent for Intervertebral Disc Regeneration. Tissue Eng A. 2018 Jun;

10. de Vries SAH, van Doeselaar M, Meij BP, Tryfonidou MA, Ito K. Notochordal cell matrix: An inhibitor of neurite and blood vessel growth? J Orthop Res. 2018 Jul;

11. Bach FC, Tellegen AR, Beukers M, Miranda-Bedate A, Teunissen M, de Jong WAM, et al. Biologic canine and human intervertebral disc repair by notochordal cell-derived matrix: From bench towards bedside. Oncotarget. 2018;9(41).

12. Xu J, Liu S, Wang S, Qiu P, Chen P, Lin X, et al. Decellularised nucleus pulposus as a potential biologic scaffold for disc tissue engineering. Mater Sci Eng Mater Biol Appl. 2019 Jun;99:1213–25.
